# Liver Fibrosis: Therapeutic Targets and Advances in Drug Therapy

**DOI:** 10.3389/fcell.2021.730176

**Published:** 2021-09-21

**Authors:** Zui Tan, Hongbao Sun, Taixiong Xue, Cailing Gan, Hongyao Liu, Yuting Xie, Yuqin Yao, Tinghong Ye

**Affiliations:** ^1^Sichuan University-University of Oxford Huaxi Joint Centre for Gastrointestinal Cancer, Frontiers Science Center for Disease-Related Molecular Network, Department of Gastroenterology and Hepatology, State Key Laboratory of Biotherapy, West China Hospital, Sichuan University, Chengdu, China; ^2^West China School of Public Health and West China Fourth Hospital, Sichuan University, Chengdu, China

**Keywords:** liver fibrosis, targets, drug therapy, hepatic stellate cells, extracellular matrix

## Abstract

Liver fibrosis is an abnormal wound repair response caused by a variety of chronic liver injuries, which is characterized by over-deposition of diffuse extracellular matrix (ECM) and anomalous hyperplasia of connective tissue, and it may further develop into liver cirrhosis, liver failure or liver cancer. To date, chronic liver diseases accompanied with liver fibrosis have caused significant morbidity and mortality in the world with increasing tendency. Although early liver fibrosis has been reported to be reversible, the detailed mechanism of reversing liver fibrosis is still unclear and there is lack of an effective treatment for liver fibrosis. Thus, it is still a top priority for the research and development of anti-fibrosis drugs. In recent years, many strategies have emerged as crucial means to inhibit the occurrence and development of liver fibrosis including anti-inflammation and liver protection, inhibition of hepatic stellate cells (HSCs) activation and proliferation, reduction of ECM overproduction and acceleration of ECM degradation. Moreover, gene therapy has been proved to be a promising anti-fibrosis method. Here, we provide an overview of the relevant targets and drugs under development. We aim to classify and summarize their potential roles in treatment of liver fibrosis, and discuss the challenges and development of anti-fibrosis drugs.

## Introduction

Liver fibrosis is an abnormal repair reaction for chronic liver injury caused by various causes, such as chronic hepatitis B (CHB), chronic hepatitis C (CHC) and alcoholic fatty liver disease (AFLD). It is characterized by diffuse excessive production and deposition of extracellular matrix (ECM) in liver (Poynard et al., 1997; Benhamou et al., 1999; Pinzani and Macias-Barragan, 2010; Povero et al., 2010). Organism initiates pro-inflammatory mechanism firstly when the injury accumulates. With the pro-inflammatory reaction, the normal structure and physiological function of the liver tissues are gradually destroyed, which causes the production of scar tissues replacing the liver parenchyma. It further develops into liver cirrhosis, liver failure or liver cancer, which eventually leads to the death of the patients (Zoubek et al., 2017).

In recent years, with the in-depth study of the occurrence and development mechanism of liver fibrosis and the use of clinical drug, it is found that cleaning pathogens or removing etiology, such as blocking or curing virus infection, has the potential of reversing liver fibrosis. Yet, there are still many great difficulties in the reversal of liver fibrosis. Although many anti-fibrotic candidate drugs have shown good results in experimental animal models, their anti-fibrotic effects in clinical trials remain very limited.

In this review, we classify and summarize the relevant targets and drugs under research and development for the treatment of liver fibrosis at home and abroad, and we also explore their potential roles and curative effects, and discuss the challenges in the research and development of anti-fibrosis drugs.

## Pathogenesis of Liver Fibrosis

**FIGURE 1:**
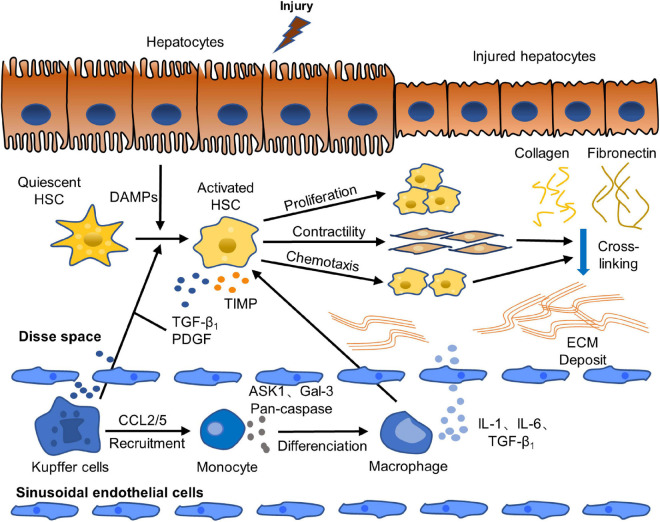
Pathogenesis of liver fibrosis. Activation of HSCs is a crucial step of the occurrence and progression of liver fibrosis. Quiescent HSCs are activated to fibrogenic phenotype by DAMPs released by injured hepatocytes. Activated HSCs are continuously activated and proliferated by paracrine and autocrine. They secret abundant fibrogenic cytokines and produce excessive ECM, which causes the break of the balance of pro-fibrosis/anti-fibrosis mechanism. The pro-fibrosis mechanism leads to the abnormal formation of scar and eventually induces liver fibrosis (Tacke and Weiskirchen, 2012; Roehlen et al., 2020).

Liver fibrosis is caused by chronic liver injuries which can be induced by virus infection, autoimmune diseases, metabolic diseases, drug toxicity, alcoholic liver disease (ALD), non-alcoholic fatty liver disease (NAFLD) and so on (de Alwis and Day, 2008; Pinzani and Macias-Barragan, 2010). With short-term liver injury, liver fibrosis will not occur due to the balance of pro-fibrosis and anti-fibrosis mechanisms. However, when a long-term or chronic liver injury occurs, the hepatocyte membrane is destroyed, which causes hepatocyte’s necrosis and apoptosis. The injured hepatocyte releases damage-associated molecular patterns (DAMPs) which stimulate the transformation of quiescent hepatic stellate cells (HSCs) into activated ones directly. And then, fibrogenic phenotype of HSCs is activated, and excessive ECM was produced with the main components of type I and III collagen and fibronectin, leading to the balance between matrix metalloproteinases (MMPs) and tissue inhibitors of metalloproteinase (TIMPs), which regulate the synthesis and degradation of ECM, to be broken. MMPs that promote ECM degradation decrease, while TIMPs that inhibit MMPs increase. The imbalance between MMPs and TIMPs leads to the excessive deposition of ECM in the Disse space and the formation of scar (Zhu et al., 2004; Tacke and Weiskirchen, 2012). The imbalance between pro-fibrosis and anti-fibrosis mechanisms results in the destruction of liver tissue structure and normal physiological function, and eventually leads to the formation of liver fibrosis. Moreover, the activated HSCs have increased contractility, express alpha smooth muscle actin (α-SMA) highly, and secrete cytokines, such as transforming growth factor beta 1 (TGF-β_1_), platelet derived growth factor (PDGF), and connective tissue growth factor (CTGF). The autocrine of activated HSCs further activates HSCs continuously. And activated HSCs also secrete chemokines, which move to the injured liver site, chemotactically accumulate in the inflammatory compartment and aggravate inflammatory damage. In addition, DAMPs released by injured hepatocytes stimulate the activation of Kupffer cells and other immune cells which further stimulate the activation of HSCs and maintain its survival via secreting pro-inflammatory and pro-fibrotic factors to induce inflammation, such as PDGF, TGF-β_1_, tumor necrosis factor alpha (TNF-α) and interleukin-1 beta (IL-1β), and activating TGF-β_1_/Smad signal pathway, mitogen-activated protein kinase (MAPK) signal pathway and other signal pathways. Furthermore, Kupffer cells also secrete chemokine (C-C motif) ligand 2 (CCL2) and CCL5, which recruit monocytes to inflammatory injured site. Moreover, monocytes further cause hepatocyte injury, promote HSCs activation and aggravate inflammation and fibrosis by synthesizing and secreting pro-inflammatory and pro-fibrogenic substances including apoptosis-signal-regulating kinase 1 (ASK1), Pan-caspase, Galectin-3 (Gal-3) and so on (Aydin and Akcali, 2018; Roehlen et al., 2020). In addition, TGF-β_1_ stimulates monocytes to differentiate into macrophages. Macrophages produce inflammatory mediators, such as IL-1 and IL-6, which promotes the aggravation of the inflammatory response and the continuous activation and survival of HSCs (Li et al., 2006). The paracrine of Kupffer cells and macrophages affects the activation of HSCs. The pathogenesis of liver fibrosis is shown in Figure 1.

## Progress in the Treatment of Liver Fibrosis

### Etiological Treatment

Chronic liver disease is a major health problem in the world, and it causes about 2 million deaths every year. Nowadays, liver cirrhosis among chronic liver diseases has become the 11th most common cause of death in the world (Asrani et al., 2019). As an early stage of liver cirrhosis, liver fibrosis is a reversible and complex pathological process caused by various chronic liver diseases, just as organ fibrosis is a feature of the progression of chronic inflammatory diseases. Therefore, it is a top priority for the treatment of liver fibrosis. Etiological treatment, that is to say, elimination of primary pathogenic factors, is the primary countermeasure of anti-liver fibrosis. If the etiologies were effectively suppressed or removed, it will reduce the persistent liver injury and have a magnificent meaning in further blocking or reversing liver fibrosis.

The main cause of chronic liver diseases and liver fibrosis is hepatitis virus infection, such as CHB and CHC. Thus, anti-hepatitis virus therapy plays an important role in the treatment of liver fibrosis. At present, the main drugs for the treatment of CHB are nucleotide analogs and interferon. Entecavir, a first-line antiviral drug, was used to treat 120 CHB patients with liver fibrosis, among whom 54 patients (45%) showed fibrosis regression after 78 weeks of antiviral treatment, indicating that liver hardness continued to decrease and liver fibrosis was alleviated after effective antiviral treatment (Wu et al., 2018). Tenofovir effectively inhibited hepatic fibrosis in 148 patients with advanced fibrosis or liver cirrhosis complicated with human immunodeficiency virus-hepatitis B virus (HIV-HBV) infection (Boyd et al., 2010). Nowadays, the most effective treatment of CHC is the use of direct-acting antiviral agents (DAAs) (Premkumar and Dhiman, 2018). One of the commonly used DAAs is epclusa, “the third generation product of Gilead,” which is the compound tablet of velpatasvir and sofosbuvir (Abramowicz et al., 2017). Sofosbuvir is a nucleoside HCV NS5B polymerase inhibitor and it was explored in a prospective study, the results of which showed that liver fibrosis score and liver hardness decreased significantly in 32 CHC patients with liver fibrosis after 12 weeks of treatment (Bernuth et al., 2016). Epclusa, as a pan-genotypic drug, is used for all 6 genotypes of hepatitis C, and its cure rate for hepatitis C is up to 98%, higher than that of sofosbuvir. However, the use of epclusa is limited by the restrictions of medical insurance. Moreover, “the fourth generation product of Gilead” vosevi, which is based on epclusa with addition of voxilaprevir was approved to be listed in China at the end of December 2019. Its treatment spectrum is wider than epclusa. It is used to treat hepatitis C patients who failed to be treated with epclusa, and the cure rate is close to 100% (Link et al., 2019). Therefore, liver fibrosis caused by these chronic diseases is treated or even reversed because of the cure of these chronic diseases, that is, the removal of the cause. In addition, autoimmune hepatitis (AIH), drug-induced liver injury (DILI), non-alcoholic steatohepatitis (NASH), and alcoholic steatohepatitis (ASH) are also the main causes of liver fibrosis (Mueller et al., 2010; Valera et al., 2011; Tacke and Weiskirchen, 2021; Wan et al., 2021). It has been reported that fibrosis and histological activity index of 54 AIH patients with liver fibrosis who received immunosuppressive therapy decreased significantly (Valera et al., 2011). The results showed that immunosuppressive therapy is an important method for reversing liver fibrosis of AIH. Moreover, the DILI patients with liver fibrosis should reduce or stop the use of the related drugs which induced liver injury and fibrosis. Also, the patients with liver fibrosis caused by NASH should balance diet, take more exercise and control weight, while patients with ASH must abstain from alcohol.

### Anti-inflammatory and Liver Protection

#### Anti-inflammatory Treatment

The occurrence and development of fibrosis is always accompanied with inflammatory response. In liver injury, Kupffer cells firstly cause injury and initiate inflammatory cascade reaction, release a variety of inflammatory factors and secrete CCL2/5 to recruit inflammatory monocytes, macrophages and lymphocytes to the injury site. Macrophages release ASK1, TNF-α and pan-caspase, which further aggravates inflammatory injury. In addition, Kupffer cells also produce cytokines, such as TGF-β_1_, PDGF, IL-6 and Gal-3, which promote the activation and proliferation of HSCs. Furthermore, peroxisome proliferators-activated receptors (PPARs), as a kind of regulatory factor against liver fibrosis, are inhibited by activated HSCs, resulting in excessive proliferation and deposition of ECM and the occurrence and development of liver fibrosis (Li and Wang, 2007; Luo et al., 2017). Moreover, chronic liver diseases are generally accompanied by increased *de novo* lipogenesis (DNL) in the liver (Tamura and Shimomura, 2005; Ameer et al., 2014). Fat is broken down into fatty acids under the action of lipase, and the excessive accumulation of fatty acids in the liver also leads to hepatotoxicity and inflammation (Ameer et al., 2014). Therefore, it is an important measure to prevent liver fibrosis by inhibiting the accumulation of fat in the liver and reducing the secretion of inflammatory cytokines and the release of apoptotic proteins. The research progress of related drugs is summarized in Table 1.

**TABLE 1:** Anti-hepatic fibrosis drugs related to inhibiting inflammation and protecting hepatocyte cells.

Anti-fibrotic mechanism	Agent	Target	Research state	NCT number	References
Anti-inflammatory	Met-CCL5	CCR5	Preclinical study	–	Berres et al., 2010
	Cenicriviroc	CCR2/5	Phase 3	NCT03028740	Friedman et al., 2016, 2018; Tacke, 2018
	Belapectin	Gal-3	Phase 3	NCT02462967	Harrison et al., 2016; Chalasani et al., 2020
	Aspirin	TLR/NF-kB	Preclinical study	–	Liu et al., 2020
	GS-0976	ACC	Phase 3	NCT02548351	Loomba et al., 2018a
	WZ66	ACC1/2	Preclinical study	–	Gao et al., 2020
	Liraglutide	GLP-1	Phase 3	NCT02654665	Armstrong et al., 2016
	Resmetirom	THR-β	Phase 2	NCT02912260	Harrison et al., 2019
Inhibition of oxidative stress	Oroxylin A	ROS	Preclinical study	–	Shen et al., 2020
	Methyl ferulic acid	NOX4/ROS	Preclinical study	–	Cheng et al., 2019
	GKT137831	NOX1/4	Phase 2	NCT03226067	Aoyama et al., 2012; Jiang et al., 2012
	Losartan	AngII	Phase 4	NCT00298714	Colmenero et al., 2009
Inhibition of hepatocyte apoptosis	VX-166	Pan-caspase	Preclinical study	–	Witek et al., 2009
	Emricasan	Pan-caspase	Phase 2	NCT02138253	Barreyro et al., 2015; Frenette et al., 2019; Gracia-Sancho et al., 2019; Harrison et al., 2020a
	Pentoxifylline	TNF-α	Phase 2	NCT02283710	Toda et al., 2009
	β-elemene	TNF-α	Preclinical study	–	Liu et al., 2011
	Selonsertib	ASK1	Phase 3	NCT03053050; NCT03053063	Loomba et al., 2018b; Harrison et al., 2020c; Yoon et al., 2020

After liver injury, the overproduction of chemokine CCL2/CCL5 is induced in livers of mouse and human, which is related to the severity of liver fibrosis. In the two models of liver fibrosis induced by carbon tetrachloride (CCl_4_) and methionine and choline-deficient diet, it was found that *Ccl5^–/–^* mice showed a reduction of HSCs activation, immune cell infiltration and liver fibrosis. Met-CCL5, an antagonist of chemokine (C-C motif) receptor 5 (CCR5), inhibited the migration, proliferation and collagen secretion of HSCs effectively, ameliorated the liver fibrosis in experimental model mice significantly, and accelerated the regression of fibrosis (Berres et al., 2010). Therefore, it is feasible to reduce experimental liver fibrosis by antagonizing CCR5. Cenicriviroc (CVC) is a dual inhibitor of CCR2 and CCR5, and it reduced the amassment and accumulation of pro-inflammatory macrophages in animal models of liver fibrosis (Lefebvre et al., 2016). In a phase II clinical trial (NCT02217475) of NASH patients with liver fibrosis, fibrosis was ameliorated in patients who were administered with 150 mg CVC for 2 years with good safety (Friedman et al., 2016). The results of a phase 2b clinical trial of 289 patients showed that there were twice as many patients with ameliorated fibrosis and no deterioration of steatohepatitis in the 150 mg CVC group as in the placebo group after one-year treatment. The safety and tolerance of CVC were comparable to placebo in Freidman’s research, and the main adverse reactions are fatigue, diarrhea, and headache (Friedman et al., 2018; Tacke, 2018). However, a phase III study (NCT03028740) of patients with advanced fibrosis and cirrhosis, aiming to assess the efficacy and safety of CVC treatment, was terminated recently due to lack of efficacy (Anstee et al., 2020). It reveals that the effectiveness of inhibiting CCR2/CCR5 in the treatment of liver fibrosis still needs more studies to verify.

Overexpression of galactose lectin significantly promoted inflammatory response and aggravated liver fibrosis. Gal-3, as a galactose lectin with immune effects, is secreted by activated Kupffer cells and macrophages in inflammatory state and participates in the pathophysiological process of liver fibrosis (Gudowska et al., 2015; Moon et al., 2018). Belapectin (GR-MD-02), as a Gal-3 inhibitor, is a complex carbohydrate drug, and it was proved to be safe and well tolerated at the maximum dose of 8 mg/kg in phase I clinical trial of NASH patients with advanced fibrosis (Harrison et al., 2016). However, a recent phase 2b multicenter placebo-controlled clinical trial (NCT02462967) of belapectin in patients with liver fibrosis, NASH, and cirrhosis showed that it was tolerated and safe in a dose of 2 mg/kg for 52 weeks compared with placebo, but had no significant effect on reduction of fibrosis or NASH scores. A high proportion of patients in the placebo group and the belapectin group had adverse reactions such as infections and gastrointestinal diseases, with the severity of grade 1 (mild) or grade 2 (moderate). Yet, Chalasani’s research showed that belapectin with dose of 2 mg/kg reduced hepatic venous pressure gradient and variceal development in NASH patients without esophageal varices (Chalasani et al., 2020). In addition, Aspirin, a classic antipyretic and analgesic, was founded that it exerted a significant anti-inflammatory effect by inhibiting IL-6 and TNF-α and reducing the number of inflammatory cells, and it also inhibited the activation and proliferation of HSCs and liver fibrosis via inhibition of toll-like receptor 4 (TLR4)/nuclear factor kappa beta (NF-κB) signal pathway. These results suggested that Aspirin is a potential effective drug for the treatment of liver fibrosis (Liu et al., 2020). IL-1β and IL-1 receptor antagonist (IL-1ra) are important mediators of chronic liver disease. IL-1ra treatment had a certain anti-fibrotic effect in the bile duct ligation-induced (BDL) mouse model of hepatic fibrosis, but it had a pro-fibrotic effect in the CCl_4_-induced mouse model of hepatic fibrosis (Meier et al., 2019). This suggests that blocking IL-1-mediated inflammation may only be selectively beneficial to liver fibrosis.

*De novo* lipogenesis plays a major role in fatty acid metabolism and is a necessary link for HSCs activation. The first step of the synthesis of DNL is catalyzed by acetyl-CoA carboxylase (ACC) as the rate-limiting enzyme. It has been reported that the inhibition of ACC decreased liver steatosis and serum fibrosis biomarkers in patients with NASH, depressed the pro-fibrotic activity of HSCs, and reduced the severe degree of liver fibrosis in diethylnitrosamine (DMN) chemical-induced liver injury model and high fat diet-induced rat model (Ross et al., 2020). An ACC small molecule inhibitor, GS-0976, was used in a phase II randomized placebo-controlled trial (NCT02856555) of 126 NASH patients with F1–F3 fibrosis. The results showed that the fibrosis marker TIMP1 declined in a dose-dependent manner in the patients who were administered 20 mg/d GS-0976 for 12 weeks, accompanied by 30% decrease in liver fat and depression of liver injury markers, but there was no change in liver hardness. In addition, GS-0976 was safe, but the plasma triglyceride level was > 500 mg/dL observed in 16 patients, which may cause atherosclerosis (Loomba et al., 2018a). The effects of GS-0976 on cardiovascular function need long-term studies to determine. The novel ACC1/2 inhibitor WZ66 was reported that it significantly improved NASH-related liver function by reducing steatosis, triglycerides and other lipids, and inhibiting the activation of Kupffer cells and HSCs in high fat diet-induced mice model (Gao et al., 2020).

Glucagon-like peptide-1 (GLP-1) directly ameliorated the state of liver fibrosis by increasing insulin release, reducing glucagon secretion, decreasing the concentration of liver enzymes and depressing hepatic steatosis. The GLP-1 analog liraglutide was carried out in a phase II randomized placebo-controlled trial (NCT01237119) of 52 patients with NASH. The liver biopsy results showed that 39% of patients with continuous administration of 1.8 mg/d liraglutide for 48 weeks had definite non-alcoholic steatohepatitis improvement without further exacerbation of liver fibrosis, while only 9% of patients in the placebo group had improvement. Furthermore, only 9% of patients in the liraglutide group had further fibrosis compared with 36% in the placebo group. Safety and tolerability of liraglutide were comparable to placebo, and the main adverse events of liraglutide group are gastrointestinal disorders, nausea and diarrhea with the severity of grade 1 or grade 2 (Armstrong et al., 2016). Resmetirom (MGL-3196) is a selective thyroid hormone receptor β agonist with oral activity, which aims to improve NASH by increasing liver fat metabolism and reducing lipo-toxicity. The results of a 36-week multicenter randomized double-blind placebo-controlled trial (NCT02912260) of 348 patients showed that resmetirom decreased liver fat content by 32.9% and 37.3%, respectively, after 12 weeks and 36 weeks treatment with a dose of 80 mg in patients with F1–F3 fibrosis, compared with 10.4% and 8.5% in the placebo group. Moreover, most adverse events were mild or moderate, such as diarrhea and nausea (Harrison et al., 2019).

#### Antioxidant Stress

Oxidative stress is an important factor in liver injury and liver fibrosis. Oxidative stress reaction (ROS) produces excessive reactive oxygen species and active free radicals in the liver, which weakens the antioxidant function and causes the increase of active free radicals in hepatocytes, the decrease of scavenging, and the destruction of hepatocyte membrane. These results affect the function of synthesis and degradation of hepatocytes, and lead to hepatocyte necrosis and apoptosis. In addition, ROS also promotes the activation of HSCs and liver fibrosis by causing peroxidation damage to Kupffer cells and neutrophils, up-regulating the gene expression of collagen type I alpha 2 in the liver, and triggering inflammation (Schwabe and Brenner, 2006; Yang et al., 2017).

Up to now, the common anti-oxidative stress and hepatocyte protection drugs include reduced glutathione, tiopronin, silymarin, *s*-allylcysteine (SAC), oroxylin A, methyl ferulic acid (MFA) and so on. Reduced glutathione protects the hepatocyte membrane from the damage of active free radicals by accelerating the scavenging of free radicals. Tiopronin can not only scavenge free radicals, but also promote hepatocyte regeneration. Silymarin, as a classical drug for repairing liver injury, inhibits the formation of lipid peroxide and stabilizes liver cell membrane. Also, it has the effects of protecting liver and anti-liver fibrosis. SAC inhibited the fibrosis process and improved the survival rate of rats with CCl_4_-induced liver fibrosis in a dose-dependent manner. Its therapeutic effect is better than that of *N*-acetylcysteine (Kodai et al., 2015). Therefore, SAC is expected to become an effective drug for the treatment of liver fibrosis. RAP-8 showed anti-fibrotic effect by inhibiting oxidative stress and promoting cell cycle arrest (Xu et al., 2019). Oroxylin A effectively alleviated liver fibrosis by clearing ROS, suppressing phosphatidylinositol 3-kinase (PI3K)/AKT/mTOR signal transduction, and inhibiting the secretion of pro-inflammatory cytokines in activated HSCs (Shen et al., 2020). MFA, a bioactive monomer, has a protective effect on liver injury. And it inhibited liver fibrosis in CCl_4_-induced rats by inhibiting TGF-β_1_/Smad and NADPH oxidase 4 (NOX4)/ROS signal pathways, down-regulating the level of procollagen type III, collagen type IV and laminin, up-regulating the ratio of MMP2/TIMP1, and inhibiting the synthesis of ECM and the activation of HSCs (Cheng et al., 2019).

Nicotinamide adenine dinucleotide phosphate (NADPH) oxidase (NOX) is a multicomponent transmembrane enzyme complex, including six subtypes of NOX1, NOX3, NOX4, NOX5, DUOX1 and DUOX2. When the cells are subjected to stimulation, NOX receives the signal to produce ROS, and then causes oxidative damage. The key cells in the liver to produce NOX are Kupffer cells and HSCs. Kupffer cells produce only NOX2, while HSCs produces NOX1, NOX 2 and NOX 4. In the process of hepatic fibrosis, NOX1, NOX 2 and NOX 4 play a key role in HSCs activation, proliferation and ECM synthesis, and NOX4 is involved in hepatocyte apoptosis (De Minicis and Brenner, 2007; Mortezaee, 2018). As a dual NOX1/4 inhibitor, GKT137831 reduced the production of ROS in HSCs both *in vitro* and *in vivo*. It significantly inhibited the formation of hepatic fibrosis and hepatocyte apoptosis in prevention or treatment groups in mice models of hepatic fibrosis induced by CCl_4_ and BDL (Aoyama et al., 2012; Jiang et al., 2012). Currently, GKT137831 is undergoing a fibrotic effect trial (NCT03226067) on patients with primary cholangitis after 24 weeks of treatment, which expected to have a satisfactory feedback. Angiotensin II (Ang II) promoted fibrosis by phosphorylating non-phagocytic NOX regulatory subunit p47 phox and inducing oxidative stress. Losartan, as an Ang II receptor blocker, ameliorated inflammation and fibrosis in 50% of the patients who were administered with 50 mg/d for 18 months in a clinical trial of 14 HCV patients with liver fibrosis (Colmenero et al., 2009). However, there is a lack of control study in this trial, and the reliability of the results needs to be further confirmed.

#### Inhibition of Hepatocyte Apoptosis

In the process of hepatic fibrosis, hepatocyte death and apoptosis are the main influencing factors of inflammation and HSCs activation. Dead hepatocytes release DAMPs to activate HSCs and Kupffer cells. Hepatocyte apoptosis activated Fas death receptor, which induced the release of apoptotic bodies, and finally leaded to fibrogenic response (Mihm, 2018). In addition, phagocytosis of apoptotic cells activated HSCs (Witek et al., 2009). Therefore, the inhibition of hepatocyte apoptosis is beneficial to inhibit inflammation, prevent the activation of HSCs and reduce liver fibrosis.

Whether it is endogenous or exogenous apoptosis pathway, the last common step of hepatocyte apoptosis is carried out by a family of cysteine-proteases termed caspases. Pan-caspase promoted apoptosis by activating apoptotic protease. Pan-caspase inhibitor VX-166 inhibited hepatocyte apoptosis, decreased hepatic steatosis, and postponed the process of fibrosis in NASH mice model, but didn’t cause significant improvement in liver injury (Witek et al., 2009). Emricasan, a small molecule pan-caspase inhibitor, significantly ameliorated liver injury and fibrosis in NASH mice model by inhibiting caspase activity and reducing hepatocyte apoptosis, ameliorating inflammatory environment and inhibiting HSCs activation (Barreyro et al., 2015). Moreover, Emricasan dramatically ameliorated fibrosis, portal hypertension and liver function by improving hepatic sinusoidal microvascular dysfunction in rats with advanced liver cirrhosis induced by CCl_4_ (Gracia-Sancho et al., 2019). Emricasan also improved liver function of patients with severe liver cirrhosis with good safety, tolerance and similar adverse reactions compared with placebo group in a 3-month multicenter phase II randomized clinical trial (NCT02230670) of 74 patients with liver cirrhosis. The new or worsening decompensation event in the placebo group was mainly ascites, while that in the emricasan group was mainly hepatic encephalopathy, which was generally caused by the patient’s original disease (Frenette et al., 2019). However, emricasan did not improve liver inflammation or fibrosis in NASH patients with F1–F3 fibrosis who received 72 weeks of 5 mg/d or 50 mg/d treatment (NCT02686762), but may cause more severe liver fibrosis and hepatocyte swelling, which due to the activation of other cell death or necrosis mechanisms (Harrison et al., 2020a).

Tumor necrosis factor alpha participates in the activation and expression of apoptotic ligand. Its inhibitor pentoxifylline prevented porcine serum-induced liver fibrosis in rats by inhibiting the production of IL-6 and the proliferation of HSCs. In addition, pentoxifylline decreased the inflammatory state, reduced oxidative stress and ameliorated the degree of liver fibrosis in patients with NASH by inhibiting the transcription of TNF-α gene, but it had no significant effect on patients with alcoholic hepatitis (Toda et al., 2009). A study showed that β-elemene prevented hepatic fibrosis by down-regulating the expression of serum TNF-α and liver CD14 and decreasing plasma endotoxin in CCl_4_-induced hepatic fibrosis rats (Liu et al., 2011).

Another way to reduce hepatocyte death associated with liver injury is to suppress stress signals. ASK1, which is activated by a variety of pro-fibrotic factors, activates MAPK signal pathway, and participates in hepatocyte apoptosis, inflammation and fibrosis. Selonsertib (GS-4997), a selective ASK1 inhibitor, inhibited HSCs proliferation and ECM production by blocking the ASK1/MAPK pathway, and significantly alleviated DMN-induced liver fibrosis in rats (Yoon et al., 2020). Selonsertib was used to treat 74 NASH patients with F2–F3 fibrosis for 24 weeks with dose of 6 mg/d or 18 mg/d in a multicenter phase II clinical trial. The results demonstrated that selonsertib decreased fibrosis-related markers and biomarkers in the process of apoptosis, reduced inflammatory levels, and ameliorated hepatic fibrosis. Most patients have experienced mild or moderate adverse reactions, such as headache, nausea and rhinitis. In addition, three patients in the selonsertib group discontinued treatment due to serious adverse reactions (Loomba et al., 2018b; Harrison et al., 2020c). Moreover, the results need to be confirmed by further studies, because the placebo control group was not included in this study. Yet, selonsertib had no anti-fibrotic effect on NASH patients with F3 or F4 fibrosis after 48 weeks treatment with dose of 6 mg/d or 18 mg/d in a phase III clinical trial (NCT03053050; NCT03053063) (Harrison et al., 2020c).

### Inhibition of Activation and Proliferation of Hepatic Stellate Cells

The activation of HSCs is a key event in the occurrence and development of liver fibrosis. Quiescent HSCs are activated after being stimulated by liver injury. HSCs are continuously activated via TGF-β_1_, PDGF, CTGF and other cytokines secreted by Kupffer cells and other cells, which promote the proliferation and prolong the survival time of HSCs through related signaling pathways. Furthermore, autocrine action of HSCs also activates itself (Aydin and Akcali, 2018). In addition, the activation of HSCs is also promoted via some proteases, such as 3-hydroxy-3-methylglutaryl-CoA (HMG-CoA) reductase and dipeptidyl peptidase-4 (DPP4). The activated HSCs, as the main source of ECM, lead to the deposition of a large number of ECM, formation of scar tissue, destruction of normal liver tissue structure and function, and the occurrence of liver fibrosis. Therefore, inhibiting the activation and proliferation of HSCs is the key to alleviate or even reverse hepatic fibrosis. The related drugs are summarized in Table 2.

**TABLE 2:** Anti-hepatic fibrosis drugs related to inhibiting HSCs activation and proliferation.

Anti-fibrotic mechanism	Agent	Target	Research state	NCT number	References
Inhibition of TGF-β_1_/Smad	Pirfenidone	TGF-β_1_	Phase 2	NCT02161952 NCT04099407	Garcia et al., 2002; Flores-Contreras et al., 2014; Seniutkin et al., 2018; Salah et al., 2019
	Fluorofenidone	TGF-β_1_	Phase 1		Peng et al., 2019
	Praziquantel	Smad 7	Preclinical study	–	Liu et al., 2019
	Ferulic acid	Smad2/3	Preclinical study	–	Mu et al., 2018
Inhibition of PDGF	Sorafenib	PDGFR	Phase 3	NCT01849588	Wang et al., 2010; Sung et al., 2018
	AZD6244	MEK	Preclinical study	–	Sung et al., 2018
	Nilotinib	PDGFR	Preclinical study	–	Shiha et al., 2014
	Rilpivirine	STAT1	Preclinical study	–	Marti-Rodrigo et al., 2020
Inhibition of CTGF	Saracatinib	CTGF	Preclinical study	–	Du et al., 2020; Seo et al., 2020
	Pioglitazone	CTGF	Preclinical study	–	Jia et al., 2007
	Curcumin	CTGF	Preclinical study	–	Chen and Zheng, 2008
Inhibition of FGF	Pegbelfermin	FGF	Phase 2	NCT02413372	Sanyal et al., 2019a; Verzijl et al., 2020
	NGM282	FGF	Phase 2	NCT02443116	Harrison et al., 2018, 2020b
	Hydronidone	FGFR1	Phase 2	NCT02499562	Liu et al., 2017
Inhibition of Wnt/β-catenin	ICG001	CBP/β-catenin	Preclinical study	–	Akcora et al., 2018
	PrI-724	CBP/β-catenin	Phase 2	NCT02195440 NCT03620474	Kimura et al., 2017; Tokunaga et al., 2017
	Octreotide	Wnt/β-catenin	Preclinical study	–	Zhang et al., 2018
Inhibition of FXR	Obeticholic acid	FXR	Phase 3	NCT02548351	Neuschwander-Tetri et al., 2015; Goto et al., 2018; Fan et al., 2019; Younossi et al., 2019
	Cilofexor	FXR	Phase 2	NCT02854605	Patel et al., 2020
	PX20606	FXR	Preclinical study	–	Schwabl et al., 2017
Inhibition of CB1R	Rimonaban	CB1R	Preclinical study	–	Giannone et al., 2012
	SR141716A	CB1R	Preclinical study	–	Teixeira-Clerc et al., 2006
	JD5037	CB1R	Preclinical study	–	Tan et al., 2020
Inhibition of PPARs	Elafibranor	PPAR-α/δ	Phase 3	NCT01694849	Ratziu et al., 2016
	Rosiglitazone	PPAR-γ	Phase 2	NCT02704403	Wei et al., 2019
Inhibition of HMG-CoA reductase	Statins	HMG-CoA reductase	Phase 2	NCT03780673; NCT02968810; NCT04072601	Oberti et al., 1997; Abraldes et al., 2009; Jang et al., 2018
Inhibition of DPP4	Sitagliptin	DPP4	Preclinical study	–	Iwasaki et al., 2011
	Alogliptin	DPP4	Preclinical study	–	Zhang et al., 2019

#### Inhibition of TGF-β_1_/Smad Signal Pathway

Tumor necrosis factor-β_1_ is a vital profibrotic cytokine in the development of liver fibrosis. The up-regulation of TGF-β_1_/Smad signal pathway is one of the most important factors in the process of liver fibrosis. During liver injury, TGF-β_1_ binds to the type II receptor on HSCs, which recruits and activates type I receptor via phosphorylation of serine residues. The activated type I receptor activates receptor-regulated protein Smad2/3 via re-phosphorylation, which separates from the receptor and forms a complex with Smad4. The complex trans-locates to the nucleus and down-regulates the expression of Smad7, which inhibits TGF-β_1_ by negative feedback, regulates the expression of fibrosis-related genes and induces the activation, proliferation and trans-differentiation of HSC into myofibroblasts, promotes the excessive synthesis and deposition of ECM and finally aggravates fibrosis (Derynck and Zhang, 2003). Therefore, the inhibition of TGF-β_1_/Smad signal pathway plays a critical role in inhibiting the activation and proliferation of HSCs and ameliorating liver fibrosis.

Pirfenidone (PFD) is a broad-spectrum anti-fibrotic drug, which was approved by FDA in 2014 for the treatment of idiopathic pulmonary fibrosis. Preclinical studies showed that PFD effectively ameliorated the liver inflammation and fibrosis induced by concanavalin A, CCl_4_ and BDL in mice by significantly reducing the level of serum TGF-β_1_ and collagen expression (Garcia et al., 2002; Seniutkin et al., 2018; Salah et al., 2019). At present, the study of PFD against liver fibrosis has entered phase II clinical trials. The results of treating 22 patients with HCV infection showed that PFD significantly ameliorated liver fibrosis in 67% of the patients with continuous administration for 2 years by significantly decreasing TGF-β_1_ levels and ameliorating inflammation, steatosis and liver function. The adverse events are mild, such as gastritis and nausea (Flores-Contreras et al., 2014). Yet, because of no placebo control group in this study, further research is needed to confirm these results. In addition, a clinical study (NCT04099407) of prolonged-release formulation pirfenidone (PR-PFD) showed that it significantly reduced fibrosis in 35% of the 122 patients who had advanced liver fibrosis with different chronic liver injury diseases and were treated for one year, by decreasing levels of TGF-β_1_ and improving liver function, while there was only 4.1% in non-PR-PFD group. Moreover, only 12% patients had transient burning or nausea and 7% patients had photosensitivity, indicating that PR-PFD had good safety in advanced liver fibrosis (Poo et al., 2020). Therefore, PR-PFD is a potential candidate drug for anti-fibrosis. The me-better drug of PFD, Fluorofenidone, alleviated liver fibrosis and liver injury induced by porcine serum in rats through reducing the activation of HSCs induced by TGF-β_1_ and inhibiting TGF-β_1/_Smad and MAPK signal pathways (Peng et al., 2019). Furthermore, praziquantel, as a schistosomicide with good safety, significantly alleviated liver fibrosis induced by CCl_4_ in mice by up-regulating the expression of Smad7 in HSCs, inhibiting TGF-β_1_/Smad signal pathway, inhibiting the activation of HSCs and reducing collagen production (Liu et al., 2019). In addition, ferulic acid effectively improved hepatic fibrosis *in vivo* and *in vitro* by inhibiting the expression of α-SMA, collagen, fibronectin and other fibrosis markers in human HSC line LX2 induced by TGF-β_1_, inhibiting the protein levels of p-Smad2 and p-Smad3 in CCl_4_-induced hepatic fibrosis model, and inhibiting TGF-β_1_/Smad signal pathway (Mu et al., 2018).

Although inhibiting TGF-β_1_/Smad signal pathway is challenging for liver fibrosis, it still has pros and cons. TGF-β_1_/Smad signal pathway is crucial to maintain liver immune homeostasis through its anti-inflammatory nature and growth regulatory function. TGF-β_1_ inhibits the activation of macrophages and expression of inflammatory factors, such as TNF-α and MMP-12, in a manner that depends on smad3, which balances the immune microenvironment (Li et al., 2006). It has been reported that the levels of TGF-β_1_ on patients with null-mild liver fibrosis are higher than those with advanced liver fibrosis, which hint that TGF-β_1_ may exert an anti-fibrotic effect through its anti-inflammatory activity and immune regulatory functions, as the improvement of liver inflammation can improve liver fibrosis in patients (Rallón et al., 2011). In addition, TGF-β_1_ regulates the proliferation, differentiation, survival and functions of various immune cells, such as T lymphocytes, B lymphocytes, dendritic cells, and macrophages. Since those immune cells play an important role in mediating liver homeostasis (Li et al., 2006), blocking TGF-β_1_ may lead to disorders of liver homeostasis. Therefore, it is crucial to focus on how to balance the relationship between the pro-fibrotic activity, anti-inflammatory activity and functions of maintaining liver homeostasis of TGF-β_1_/Smad.

#### Inhibition of PDGF Receptor-Mediated Signaling Pathway

Platelet derived growth factor is an important mitogen in the differentiation of HSCs. During liver injury, Kupffer cells mediate platelet recruitment in the liver and produce a large number of PDGF. In addition, endothelial cells and activated HSCs also express PDGF. The binding of PDGF to its receptor (PDGFR) induces dimerization and phosphorylation of the receptor, which in turn phosphorylates tyrosine residues on different substrates in the cell. These results regulate the expression level of fibro-genic target genes, such as collagen type I alpha 1 (COL1a1), TIMPs, MMPs and apoptosis regulatory factor B cell lymphoma/lewkmia 2 (Bcl2), which leads to the survival and proliferation of HSCs (Borkham-Kamphorst and Weiskirchen, 2016). Stimulation of PDGFR activates several signal pathways, including Ras/extracellular signal-regulated protein kinase/MAPK pathway, PI3K/AKT pathway, Janus kinase/signal transducer and transcriptional activator (STAT) pathway. Moreover, PDGFR mRNA deletion in hepatocytes inhibited the up-regulation of PDGFR mRNA expression in HSCs, decreased activation of HSCs and alleviated liver fibrosis (Lim et al., 2018). Therefore, the inhibition of PDGFR is useful to inhibit the proliferation of HSCs and alleviate liver fibrosis.

A study founded that sorafenib up-regulated the expression of Fas, Fas-L and Caspase-3 by inhibiting PDFGR and VEGFR2, decreased the ratio of Bcl2 to Bcl2-related protein x (Bax), inhibited the proliferation of HSCs, promoted apoptosis of HSCs, reduced collagen accumulation, and alleviated liver fibrosis (Wang et al., 2010; Sung et al., 2018). However, another study founded that although sorafenib inhibited the levels of PDFGR and p-AKT when HSCs were treated with low-dose sorafenib with sub-micromolar concentration, it still induced the activation of MAPK in HSCs and promoted the differentiation of myofibroblasts. Sorafenib and MEK inhibitor AZD6244 jointly inhibited the contradictory activation of MAPK and HSCs *in vitro* through chemokine (C-X-C motif) receptor 4-targeted nanoparticles delivery, and alleviated liver fibrosis in liver injury model induced by CCl_4_ in mice (Sung et al., 2018). Nilotinib, an inhibitor of tyrosine kinase, down-regulated the level of pro-fibrosis cytokines by reducing the expression of PDGFR and the level of TGF-β_1_, and significantly reduced CCl_4_-induced hepatic fibrosis in rats (Shiha et al., 2014). Dihydroartemisinin promoted the activation of caspase cascade in HSCs, up-regulated Bax, and down-regulated Bcl, suppressed PI3K/AKT pathway, inhibited proliferation of HSCs and induced its apoptosis, improved liver tissue structure, and ameliorated liver fibrosis induced by BDL in rats (Chen et al., 2016). Asiatic acid inhibited the activation of HSCs and the synthesis of ECM by reducing oxidative stress, inflammation and hepatocyte apoptosis, and inhibited PI3K/AKT/mTOR signal pathway, which effectively improved CCl_4_-induced liver injury and fibrosis in rats (Wei et al., 2018). Rilpivirine (RPV) is an anti-HIV drug with no hepatotoxicity reported. It reduced collagen expression *in vitro*, and had obvious anti-inflammatory and anti-fibrosis effects in NAFLD model and rat model of liver fibrosis induced by CCl_4_ and BDL. Through selective activation of STAT1, RPV promoted STAT3-dependent hepatocyte proliferation and HSCs apoptosis, and had bystander effects on hepatocytes, which promoted liver regeneration and ameliorated liver fibrosis (Marti-Rodrigo et al., 2020).

#### Inhibition of CTGF

Connective tissue growth factor is an important pro-fibrotic factor in the process of fibrosis, and is induced by TGF-β_1_. It promotes the production of ECM and enhances the proliferation, migration and survival ability of activated HSCs, which promotes the occurrence and development of liver fibrosis in different liver chronic diseases (Kovalenko et al., 2009).

It has been reported that pioglitazone inhibited the development of liver fibrosis by inhibiting the expression of CTGF and type III collagen in HSCs and preventing the morphological changes of HSCs induced by TGF-β_1_ in a dose-dependent manner (Jia et al., 2007). Curcumin ameliorated fibrosis by inhibiting the expression of CTGF, preventing the activation of HSCs *in vitro*, and reducing the synthesis of ECM (Chen and Zheng, 2008). Moreover, basic studies have shown that CTGF small interfering RNA (siRNA) significantly inhibited the expression of CTGF, type I collagen, type III collagen and hyaluronic acid, reduced the synthesis and secretion of ECM, alleviated liver fibrosis and protected liver function (Li et al., 2004). Additionally, Src family kinases (SFKs), as non-receptor tyrosine kinases, were activated by CTGF induced by TGF-β_1_ and had an essential effect on transcription of CTGF (Zhang et al., 2010). The expression of Src kinases is up-regulated in mice with liver fibrosis and cirrhosis induced by thioacetamide (TAA), and the expression of phosphorylated Src kinases is up-regulated when HSC is activated. Saracatinib, an inhibitor of Src kinases, attenuated the expression of type I collagen, CTGF and α-SMA in mice induced by TAA. What’s more, inhibition of Src kinases increased autophagy flux and reduced liver fibrosis (Seo et al., 2020). SU6656, a dual inhibitor of Src family and Aurora kinases, reduced CTGF expression by inhibiting Src kinases in non-transformed epithelial cells (Cicha et al., 2014). As a member of SFK, Fyn was activated in the liver of patients with fibrosis and knockdown *Fyn* with siRNA or gene knockout significantly prevented the activation of HSCs and reduced fibrosis in CCl_4_-induced mice. Saracatinib treatment decreased the activation of Fyn, prevented the activation of HSCs, and depressed the severity of liver fibrosis in mice induced by CCl_4_ (Du et al., 2020).

#### Inhibition of Fibroblast Growth Factor

There are several isoforms in the Fibroblast Growth Factor (FGF) family, which bind to four different receptors (FGFR1-4). Among them, FGF15/19 and FGF21 could inhibit the occurrence of liver fibrosis by down-regulating HSCs activation. In addition, FGFR1-mediated signal transduction is closely related to hepatic fibrosis and liver cirrhosis. FGF21 inhibits the activation of HSCs by down-regulating the expression of TGF-β_1_, decreasing the phosphorylation level of Smad2/3 and reducing the nuclear translocation of NF-κB. FGF21 also induces apoptosis of activated HSCs by increasing the expression of caspase 3 and decreasing the ratio of Bcl2 to Bax (Xu et al., 2016).

Pegbelfermin (BMS-986036) is a polyethylene glycol modified FGF21 analog. In a multicenter double-blind phase 2a clinical trial (NCT02413372), 75 NASH patients with F1–F3 fibrosis were treated with 10 mg or 20 mg pegbelfermin once a day for 16 weeks. The results showed that the absolute liver fat fraction in the pegbelfermin group was much lower than that in the placebo group, and most of the adverse events were mild, such as ascites and varicose, indicating that pegbelfermin ameliorated NASH, steatosis, liver injury and fibrosis (Sanyal et al., 2019a; Verzijl et al., 2020). FGF19 is a hormone that regulates the synthesis of bile acids directly in the liver. The engineered FGF19 analog NGM282 was injected subcutaneously with 3 mg or 6 mg to 82 patients with F1–F3 fibrosis for 12 weeks in a randomized, double-blind, placebo-controlled phase II clinical trial (NCT02443116). The results indicated that the absolute liver fat contents were at least 5% lower than the baseline in 20 patients (74%) in the 3 mg group and 22 (79%) patients in the 6 mg group while in only 2 patients (7%) in the placebo group (Harrison et al., 2018). This study demonstrated that NGM282 played a positive role in improving the condition of patients with NASH. In another open label study, NGM282 with 1 mg or 3 mg administration for 12 weeks improved the histological characteristics of NASH, reduced fibrosis-related markers significantly, decreased NASH and fibrosis scores, and ameliorated fibrosis effectively. And the most common adverse reactions are mild or moderate abdominal pain, diarrhea and nausea (Harrison et al., 2020b). The FGFR1 inhibitor hydronidone improved the inflammation and fibrosis in rats with hepatic fibrosis induced by CCl_4_, DMN and human serum albumin. Hydronidone was well tolerated with no obvious adverse reaction in phase II clinical trial (NCT02499562), but its absorption rate and degree decreased via food intake (Liu et al., 2017).

#### Inhibition of Wnt/β-Catenin Signal Pathway

Studies have shown that Wnt/β-catenin signal pathway is related to the activation of HSCs and hepatic fibrosis. Wnt protein forms a ternary complex with frizzled receptor and lipoprotein receptor-related protein (LRP)-5/6, which blocks the degradation of β-catenin. β-catenin is activated with the help of coactivators such as cyclic-AMP response element binding protein binding protein (CBP), and then accumulates and easily locates in the nucleus, which activates the transcription of related target genes (Ge et al., 2014). During liver injury, Wnt/β-catenin signal pathway is abnormally activated in activated HSCs and alleviates liver fibrosis by promoting collagen deposition and epithelial-mesenchymal transition (EMT) (Nishikawa et al., 2018).

ICG001 is a small molecular inhibitor that disrupts the interaction between CBP and β-catenin. ICG001 reduced the secretion of CCL12 and prevented the infiltration of macrophages by inhibiting the Wnt/β-catenin signal pathway in HSCs, which reduced liver inflammation and significantly decreased the activation of HSCs and the accumulation of ECM in liver fibrosis model induced by CCl_4_ in mice (Akcora et al., 2018). CBP/β-catenin inhibitor PRI-724 improved HCV-induced liver fibrosis in mice by inhibiting the activation of HSCs (Tokunaga et al., 2017). In a single-center phase I clinical trial (NCT02195440), PRI-724 was well tolerated in HCV cirrhotic patients who were given 10 mg/d or 40 mg/d within 12 weeks. However, severe liver injury may occur in cirrhotic patients with HCV given 160 mg/d PRI-724 (Kimura et al., 2017). At present, a phase 1/2a clinical trial (NCT03620474) of PRI-724 in patients with hepatitis B or hepatitis C-related liver cirrhosis is under way, which is expected to have some implications for the study of PRI-724 in liver fibrosis. Octreotide is an analog of somatostatin. It significantly inhibited the expression of Wnt1 and β-catenin *in vitro* and *in vivo*, depressed the activation and proliferation of LX2 and reduced CCl_4_-induced liver fibrosis in rats (Zhang et al., 2018). These findings provide more options for the treatment of liver fibrosis.

#### Inhibition of Farnesoid-X Receptor

Farnesoid-X receptor (FXR) is an inherent inhibitor of apoptosis in hepatocytes. It interacts with caspase-8 in the cytoplasm, prevents the formation of death-induced signal complex and the activation of caspase-8, mediates the inhibition of HSCs activation and ameliorates liver fibrosis (Wang et al., 2018). Some studies found that the lack of FXR aggravated liver fibrosis and inflammation in mice, indicating that FXR played a key role in protecting the liver from inflammation and fibrosis (Ferrell et al., 2019). Obviously, FXR is a very important anti-fibrosis target.

Farnesoid-X receptor agonist obeticholic acid (OCA, INT-747) is a semisynthetic chenodeoxycholic acid, which has good anti-fibrotic activity in animal model of liver fibrosis (Goto et al., 2018; Fan et al., 2019). In 2015, a double-blind, randomized placebo-controlled phase 2b clinical trial of 283 NASH patients showed that liver histology improved significantly in 45% patients after 72 weeks of short-term 25 mg/d OCA treatment, compared with 21% in the placebo group. But 23% patients in the OCA group had adverse reactions such as itching, compared with only 6% in the placebo group. These results indicate that the safety of OCA needs further research to determine (Neuschwander-Tetri et al., 2015). Recently, the mid-term results of the first 18 months of a multicenter, randomized placebo-controlled phase III clinical trial (NCT02548351) of 931 NASH patients with F2–F3 fibrosis who received long-term treatment with OCA showed that 23% of the patients in the 25 mg OCA group had a significant improvement on the severe degree of fibrosis and NASH in a dose-dependent manner, compared with 12% in the placebo group. And the most common adverse reaction was pruritus (Younossi et al., 2019). Cilofexor (GS-9674) is a small non-steroidal agonist of FXR. In a double-blind placebo-controlled phase II clinical trial (NCT02854605), 140 patients with NASH were administered with 30 mg or 100 mg cilofexor for 24 weeks. The results showed that cilofexor significantly reduced hepatic steatosis and serum bile acid in NASH patients and was well tolerated. Moderate to severe itching was more common in the 100 mg cilofexor group (14%) than that in the 30 mg group (4%) and the placebo group (4%) (Patel et al., 2020). In another study, FXR agonist PX20606 effectively improved liver fibrosis in CCl_4_-induced cirrhotic rats by reducing the expression of collagen (Schwabl et al., 2017).

#### Inhibition of Cannabinoid Receptor 1

Endogenous cannabinoid system is involved in the pathogenesis of liver fibrosis. In normal liver, the expression of cannabinoid receptor is very low. However, in ALD, NAFLD, liver regeneration/injury, liver fibrosis/cirrhosis and liver cancer, cannabinoid receptor 1 (CB1), a kind of G protein-coupled receptor, is up-regulated in liver myofibroblasts due to extracellular stimulation, which promotes the development of liver fibrosis (Teixeira-Clerc et al., 2006; Bataller and Gao, 2013). Therefore, blocking the CB1 signal pathway is expected to become a new strategy to treat a variety of liver diseases including liver fibrosis.

Rimonaban, a CB1 receptor (CB1R) antagonist, significantly reduced inflammation and fibrosis in CCl_4_-induced cirrhotic rats by down-regulating the expression of fibrosis and inflammation-related genes. It showed that even in the late stage of the disease, pharmacological CB1 antagonism still had a positive effect on the regression of fibrosis (Giannone et al., 2012). Another CB1R antagonist SR141716A effectively alleviated hepatic fibrosis in three chronic liver injury models by inhibiting CB1 to decrease the expression of TGF-β_1_ and prevent the accumulation of fibrogenic cells in the liver (Teixeira-Clerc et al., 2006). JD5037 is a peripheral CB1 antagonist. It attenuated CB1R-regulated activation of HSCs and liver fibrosis by inhibiting CB1R-arrestin1/AKT signal pathway, as CB1R, which were induced in liver sections of patients and mice with hepatic fibrosis, promoted the activation of HSCs by recruiting beta arrestin1 and activating AKT signal pathway. Therefore, JD5037 is a potential compound for anti-hepatic fibrosis (Tan et al., 2020).

#### Activation of Peroxisome Proliferator Activated Receptors

Peroxisome proliferators-activated receptors are a family of nuclear receptors, including PPAR-α, PPAR-β/δ and PPAR-γ. PPAR-α is a critical regulatory factor against hepatic fibrosis. PPAR-γ negatively regulates the activity of HSCs in hepatic fibrosis and reduces the differentiation of myofibroblasts (Wei et al., 2019).

Elafibranor (GFT-505) is a PPAR-α/δ agonist. In a randomized placebo-controlled phase II clinical trial (NCT01694849) involving 276 patients with NASH, the results of continuous treatment with 120 mg elafibranor for 52 weeks indicated that elafibranor ameliorated liver fibrosis and liver function while improving the NASH status of the patients, and was well tolerated. However, in the intended treatment, there was no significant difference between the elafibranor group and the placebo group. Elafibranor caused a slight and reversible increase in creatinine levels, but it did not cause adverse effects on patients with renal insufficiency (Ratziu et al., 2016). Rosiglitazone, a PPAR-γ agonist, improved BDL-induced liver fibrosis in mice by regulating NF-κB-TNF-α pathway in a PPAR-γ-dependent manner, down-regulating the expression of TGF-β_1_, α-SMA and type I collagen, inhibiting NF-κB phosphorylation, and alleviating inflammation, but it did not alleviate liver injury in Hep *Ppar*-γ KO mice (Wei et al., 2019). 15-d-PGJ2 (PPAR-γ natural ligand) or GW7845 (synthetic ligand) significantly promoted the transformation of TGF-β_1_-induced activated HSCs to quiescent phenotype by inhibiting PPAR-γ-dependent CTGF expression at both mRNA and protein levels in HSCs (Sun et al., 2009). Crocin, a naturally occurring carotenoid, was reported that it ameliorated CCl_4_-induced hepatic fibrosis in a dose-dependent manner by up-regulating the expression of PPAR-γ and down-regulating the expression of inflammatory and hepatic fibrosis-related factors (Chhimwal et al., 2020).

#### Inhibition of HMG-CoA Reductase

Statins are HMG-CoA reductase inhibitors, which reduce serum cholesterol levels by inhibiting the activity of HMG-CoA reductase. The effects of statins on reducing liver inflammation, oxidative stress and fibrosis have been reported in several studies on animal models of liver fibrosis (Oberti et al., 1997; Jang et al., 2018). Yet, the safety of statins in patients with chronic liver disease and cirrhosis needs to be evaluated in further research, considering that statins may increase the risk of rhabdomyolysis due to impaired liver CYP3A4 metabolism. Moreover, a study reported that 3% of cirrhotic patients who were administrated with statins had severe rhabdomyolysis (Abraldes et al., 2009). Currently, three placebo-controlled trials about statins (NCT03780673; NCT02968810; NCT04072601) are under way with an extended safety assessment.

#### Inhibition of Dipeptidyl Peptidase-4

Dipeptidyl peptidase-4 is a serine protease widely expressed on various cell surfaces, which has an influence on fibronectin-mediated interaction between hepatocytes and ECM, and participates in the adhesion of cells to collagen. In addition, DPP4 is expressed on the surface of activated HSCs. It has been reported that the DPP4 inhibitor sitagliptin ameliorated NAFLD (Iwasaki et al., 2011). Alogliptin, which is a classical DPP4 inhibitor, inhibited the activation of LX2 induced by TGF-β_1_ stimulation *in vitro*. Chronic treatment with alogliptin reduced hepatic steatosis in mice and protected them from liver injury in the hepatic fibrosis model induced by CCl_4_, which delayed the progression of hepatic fibrosis. Alogliptin also had a positive effect on ameliorating liver fibrosis via the negative regulation of HSCs activation (Zhang et al., 2019). This indicates that alogliptin is also a potential candidate drug for treatment of liver fibrosis.

### Inhibition of ECM Production and Promotion of ECM Degradation

Diffuse excessive production and deposition of ECM is the main manifestation of liver fibrosis. The production and degradation of ECM are in a relative balance under normal circumstances. However, during liver injury, activated HSCs are the main cells that produce ECM, resulting in excessive ECM production and continuous deposition. In addition, the main composition of ECM also changes from type IV and VI collagen to type I and III collagen, which increases the density and hardness of ECM, making it difficult for ECM to be degraded by protease (Iredale et al., 2013). Therefore, inhibition of ECM production and promotion of ECM degradation are two essential means of direct anti-fibrosis. The related drugs are summarized in Table 3.

**TABLE 3:** Anti-hepatic fibrosis drugs related to inhibiting ECM.

Anti-fibrotic mechanism	Agent	Target	Research state	NCT number	References
regulation of	Halofuginone	TIMPs	Preclinical study	-	Bruck et al., 2001; Liang et al., 2013
MMPs/TIMPs	FR(EtOH)	MMPs/TIMPs	Preclinical study	-	Peng et al., 2010
	BMS986263	Hsp47	Phase 1b/2	NCT02227459	Kavita et al., 2019
inhibition of LOX	Simtuzumab	LOXL2	Phase 2b	NCT01707472 NCT01672853	Meissner et al., 2016; Sanyal et al., 2019b

#### Matrix Metalloproteinases/Tissue Inhibitors of Metalloproteinase

Matrix metalloproteinases/TIMPs are important enzymes that regulate the deposition and degradation of ECM. MMPs are the main ECM-degrading enzyme in the liver and their endogenous inhibitors are TIMPs. MMP2 and MMP14 are highly expressed in activated HSCs. MMPs degrade ECM under normal circumstances. But with liver injury, MMPs are inhibited by TIMPs, which are high expressed. This causes the break of the balance between deposition and degradation of ECM, which leads to excessive deposition of ECM and eventually leads to liver fibrosis (Iredale et al., 2013). Therefore, up-regulation of MMPs or down-regulation of TIMPs activity is an effective measure to alleviate liver fibrosis.

Halofuginone, a small molecular derivative of quinolones, inhibited the expression and synthesis of collagen, reduced the level of TIMPs and alleviated hepatic fibrosis in rats induced by TAA and Con A (Bruck et al., 2001; Liang et al., 2013). It has been reported that Fraxinus rhynchophylla ethanol extract (FR(EtOH)) had an anti-fibrosis effect in CCl_4_-induced liver fibrosis in SD rats. FR(EtOH) effectively alleviated liver lesions and fibrous connective tissue proliferation by down-regulating the expression of MMP2, MMP9 and TIMP1 (Peng et al., 2010). As the main composition of ECM is type I and III collagen after liver injury and type I collagen is the most abundant collagen in fibrotic liver, inhibition type I or III collagen production and accumulation will be beneficial to the treatment of liver fibrosis. It has been reported that liposome COL1a1 siRNA specifically inhibited collagen production and accumulation in liver fibrosis model in mice (Jimenez et al., 2015). Hsp47, a kind of type I collagen molecular chaperone, has the ability to block collagen synthesis. Furthermore, Hsp47 siRNA containing vitamin A-coupled liposomes had significant anti-fibrosis effect in three liver fibrosis models *in vivo* (Sato et al., 2008). BMS986263, a kind of Hsp47 siRNA, which delivered lipid nanoparticles, did not show any toxicity to healthy people (Kavita et al., 2019). And its phase Ib/2 dose increment study (NCT02227459) has been completed recently.

#### Lysyl Oxidase

The Lysyl oxidase (LOX) family promotes the deposition of ECM by increasing the cross-linking of collagen. Therefore, inhibiting the LOX family is beneficial to reduce the deposition of ECM and alleviate liver fibrosis.

Simtuzumab (GS-6624), an antibody of lysyl oxidase-like protein 2 (LOXL2), decreased the stability of ECM by antagonizing the collagen cross-linking induced by LOXL2, and had a good therapeutic effect on liver cirrhosis and fibrosis induced by NASH. Moreover, simtuzumab was well tolerated in a 22-week phase II clinical trial (NCT01707472) of 18 patients with advanced fibrosis. The most common adverse reactions of simtuzumab treatment are fever, headache, glossitis, etc., which are mild. In addition, one patient experienced a serious adverse reaction and recovered after antibiotic treatment (Meissner et al., 2016). However, a phase Ib clinical trial (NCT01672853) of 234 patients showed that simtuzumab had no effect on preventing the progression of liver fibrosis in patients with primary sclerosing cholangitis with a dose of 75 mg or 125 mg for 96 weeks (Sanyal et al., 2019b). In addition, A study showed that the expression of LOXL2 decreased rapidly after liver injury compared with the stable up-regulation of LOX and LOXL1, indicating that LOXL2 had little effect in liver fibrosis (Perepelyuk et al., 2013). Future research should solve this problem of selectivity by specifically targeting LOX1.

### Gene Therapy

Liver transplantation is considered to be the only effective treatment for end-stage liver fibrosis, but it has some shortcomings, such as difficulty in finding liver source, immune rejection, poor prognosis and so on. In recent years, gene therapy, such as antisense oligonucleotide chain, RNA intervention and decoy oligonucleotides, is expected to solve these problems.

RNA intervention (RNAi) is a technique that uses siRNA of 21–23 nucleotides to specifically knock out target genes (Buchman, 2005). It has been reported that the direct knockout of TGF-β_1_ via siRNA significantly reduced the expression of α-SMA and type I collagen in HSC-T6 cells, and played an anti-fibrotic role in mice and rats with CCl_4_-induced hepatic fibrosis (Cheng et al., 2009). Histone deacetylase 2 (HDAC2) is an up-regulated protein found in HSCs treated with TGF-β_1_ or in fibrotic liver tissues induced by CCl_4_. Blocking the expression of HDAC2 with siRNA decreased the expression of α-SMA and COL1a1 in HSC-T6 cells treated with TGF-β_1_ (Li et al., 2016). β-Catenin siRNA inhibited collagen synthesis and β-catenin expression in HSCs in a time-dependent manner, down-regulated Wnt/β-catenin signal pathway, inhibited the proliferation and induced the apoptosis of HSC-T6 cell, which prevented the progression of hepatic fibrosis (Ge et al., 2014). It suggests that β-catenin siRNA provide a new strategy for the treatment of liver fibrosis. Lentivirus-mediated CB1 siRNA (CB1-RNAi-LV) significantly inhibited the expression of CB1 and the activation and proliferation of HSCs *in vitro*. CB1-RNAi-LV alleviated DMN-induced liver fibrosis in rats by inhibiting TGF-β_1_/Smad signal pathway, reducing the expression of α-SMA and improving EMT (Chen et al., 2012). The selective inhibition of CB1 by siRNA provides a new choice for the treatment of liver fibrosis.

In addition to siRNA-based treatment, microRNA (miRNA) is also another treatment method for liver fibrosis. MiRNA is a kind of endogenous non-coding small RNA, which regulates the expression of RNA after transcription. Miravirsen (SPC3649), which is a mixture of nucleic acid and DNA, effectively inhibited the function of miR-122 and reduced liver fibrosis in a phase 2a study (NCT01200420) of HCV infection (Zeng et al., 2015). MiR-101, a small non-coding RNA that regulates the MAPK response, significantly improved the liver function of mice with hepatic fibrosis induced by CCl_4_. MiR-101 markedly reduced the damage of liver parenchyma and postponed liver fibrosis by inhibiting the levels of α-SMA and COL1a1, reducing the accumulation of ECM components and inhibiting PI3K/AKT/mTOR signal pathway (Lei et al., 2019). MiR-29b, a small non-coding RNA downregulated in fibrotic liver tissues and in primary activated HSCs, effectively suppressed the expression of Smad3 in HSC line LX1 and decreased the expression of α-SMA and type I collagen in mice with hepatic fibrosis induced by CCl_4_. Moreover, MiR-29b dramatically prevented the progress of liver fibrosis by depressing the activation of HSCs and inhibiting the apoptosis of HSCs induced by PI3K/AKT pathway (Wang et al., 2015). The related siRNA and mRNA are summarized in Table 4.

**TABLE 4:** siRNA and mRNA for the treatment of hepatic fibrosis.

Anti-fibrotic mechanism	Target pathway/protein	Research state	NCT number	References
siRNA	TGF-β_1_	Preclinical study	–	Cheng et al., 2009
	HDAC2	Preclinical study	–	Li et al., 2016
	β-catenin	Preclinical study	–	Ge et al., 2014
	CB1	Preclinical study	–	Chen et al., 2012
miRNA	Miravirsen(miR-122)	Phase 2a	NCT01200420	Zeng et al., 2015
	miR-101	Preclinical study	–	Lei et al., 2019
	miR-29b	Preclinical study	–	Wang et al., 2015

## Discussion

**FIGURE 2:**
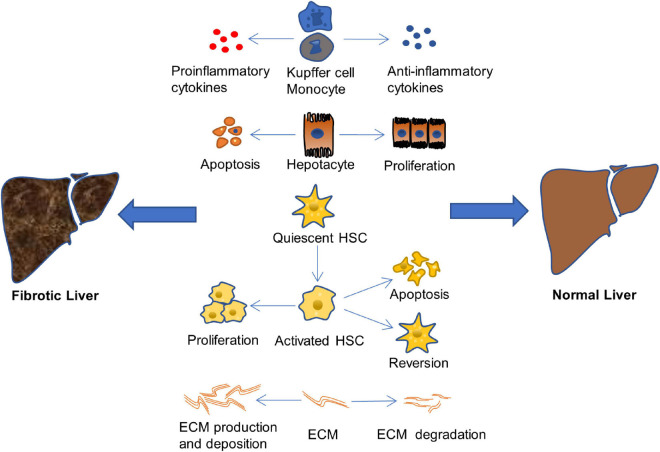
Therapeutic approaches of liver fibrosis. The liver fibrosis is induced when the balance of pro-inflammatory/anti-inflammatory, or apoptosis/proliferation of hepatocyte and HSCs, or production and deposition/degradation of ECM is destroyed (Campana and Iredale, 2017; Roehlen et al., 2020).

The mechanism of hepatic fibrosis formation is complex. The treatment of hepatic fibrosis aims at many links in its pathogenesis to reduce or even reverse hepatic fibrosis, mainly from the aspects of anti-inflammation and liver protection, inhibition of the proliferation and activation of HSCs, and depression of the production and deposition of ECM, as shown in Figure 2 (Campana and Iredale, 2017; Roehlen et al., 2020).

Many causes will lead to the imbalance of pro-fibrosis/anti-fibrosis mechanism and promote the occurrence and development of liver fibrosis, such as the excessive production and secretion of pro-inflammatory cytokines, the increase of hepatocyte apoptosis, the proliferation of activated HSCs, and the excessive production and deposition of ECM. On the other hand, a lot of factors effectively inhibit the occurrence and development of liver fibrosis, delay the process of fibrosis, and even reverse fibrosis and return the structure and function of the liver to normal, including the production and release of anti-inflammatory cytokines, the proliferation of hepatocyte, the apoptosis and restoration of resting phenotype of activated HSCs, as well as the increase of ECM degradation. Therefore, finding a drug that has the ability to balance pro-fibrosis/anti-fibrosis mechanism is crucial for treatment of liver fibrosis.

To date, the anti-hepatic fibrosis candidate drugs that are at the forefront of research with a good development momentum mainly include OCA, resmetirom, CVC, selonsertib, and elafibranor. Among them, OCA is very promising as a selective FXR agonist. The results of its phase II clinical trial showed that 25 mg/d OCA significantly improved liver fibrosis, steatosis and lobular inflammation with good tolerated (Neuschwander-Tetri et al., 2015). These effective results prompted OCA to enter phase III clinical trial, aiming to evaluate its long-term efficacy, clinical benefits and safety through a 7-year treatment period. The 18-month mid-term results of the phase III clinical trial showed that 25 mg/d OCA had a significant anti-liver fibrosis effect without worsening the NASH-related symptoms, and had basically the same serious adverse reactions as the placebo group (Ratziu et al., 2019; Younossi et al., 2019). In addition, OCA has good safety based on the 18-month mid-term study on health-related quality of life. OCA treatment had adverse reaction of mild itching in the early stage, which did not worsen with the treatment progresses, had better curative effects than the placebo group, and improved the quality of life of patients (Younossi et al., 2021). Therefore, OCA has a good effect in terms of efficacy, safety and quality of life based on the currently known results. In addition, clinical trials of OCA include patients with F1–F3 liver fibrosis and advanced NASH (Younossi et al., 2019). This is of great significance for researching different disease states to discover the clinical benefits of OCA, such as the discovery of biomarkers at different stages of the disease. Resmetirom is a selective thyroid hormone receptor β agonist. Its phase II clinical trial showed that it improved the symptoms of NASH patients with liver fibrosis and reduced liver toxicity by reducing fat content (Harrison et al., 2019). Its phase III clinical trial for patients with F2–F3 liver fibrosis is currently underway, and no results have been obtained yet. In addition to OCA and resmetirom, CVC is also one of the fast-developing anti-liver fibrosis candidate drugs. CVC is a dual inhibitor of CCR2/5. Its Phase II clinical trial results showed that 150 mg/d CVC improved liver fibrosis, prevented liver cirrhosis, and reduced mortality from liver-related diseases by improving NAS-related symptoms. It also has good safety and resistance (Friedman et al., 2016). However, the role of CVC treatment is mainly focused on patients with F2–F3 liver fibrosis, that is, patients with a higher risk of intermediate and advanced liver cirrhosis, and the role of CVC treatment in patients with mild liver fibrosis is still unclear. In addition, the effects of CVC on liver fibrosis are related to the reduction of inflammation-related biomarkers, such as IL-6, IL-1β, etc., which indicates that CVC is a great potential anti-liver fibrosis candidate drug because inflammation is one of the important factors causing liver fibrosis (Friedman et al., 2016). However, the results of phase III clinical part 1 of AURORA research showed that CVC has a lack of efficacy, which led to the termination of the study (Anstee et al., 2020). Fortunately, a phase II clinical trial on CVC and tropifexer (an FXR agonist) showed that the combination of CVC and tropifexer effectively ameliorated liver fibrosis (Pedrosa et al., 2020). In addition, considering that the half-life of CVC as long as 30–40 h, it has good safety for advanced liver fibrosis (Friedman et al., 2016). Therefore, the combination may be a better way for CVC to exert its efficacy. Selonsertib is a selective ASK1 inhibitor, and its phase II clinical trial showed that 18 mg/d selonsertib effectively reduced fibrosis in patients with F2–F3 liver fibrosis (Loomba et al., 2018b). However, its phase III clinical trial for patients with F3 liver fibrosis was terminated due to lack of efficacy (Harrison et al., 2020c). Moreover, a phase III clinical trial about another anti-liver fibrosis candidate drug elafibranor was also terminated, because it did not reach the alternative efficacy endpoint. It should be considered whether it is due to targeting or pathological barriers and so on. Perhaps the combination and improvement of delivery systems are important means to increase efficacy for anti-liver fibrosis candidate drugs.

Although many anti-fibrotic candidate drugs have shown good efficacy in experimental animal models, their anti-fibrotic effects in clinical trials are very limited. This may be due to the complicated pathological mechanism of liver fibrosis, which is the repair response after liver injury that whole body participates in, while most of the currently developed drugs are targeted at a single target rather than multiple targets. Moreover, the actual pathological conditions between animal models and patients have a great difference, which also leads to poor efficacy of drugs in clinical trials. In addition, obvious adverse reactions induced by large dosage are also one of main causes. In this case, gene therapy, which targets specific genes accurately, shows unique advantages of improving therapeutic effect and reducing side effects. Therefore, gene therapy is believed to be a promising direction of anti-liver fibrosis strategy in the future.

To date, most of anti-fibrosis drugs are still in the stage of preclinical research, including drugs for chronic liver disease-related liver fibrosis induced by different etiologies. There are also some drugs with clear anti-fibrosis effect, good safety and tolerance in the clinical research stage. It is believed that with the in-depth study on the pathogenesis of liver fibrosis and the continuous progress in the research and development of new drugs, the reversal of liver fibrosis will eventually become possible.

## Author Contributions

TY, YY, and ZT designed the structure of the article and revised the manuscript. ZT, HS, and CG drafted the initial manuscript and prepared the figures. HS, ZT, TX, HL, and YX revised the manuscript. All authors contributed to the article and approved the submitted version.

## Conflict of Interest

The authors declare that the research was conducted in the absence of any commercial or financial relationships that could be construed as a potential conflict of interest.

## Publisher’s Note

All claims expressed in this article are solely those of the authors and do not necessarily represent those of their affiliated organizations, or those of the publisher, the editors and the reviewers. Any product that may be evaluated in this article, or claim that may be made by its manufacturer, is not guaranteed or endorsed by the publisher.

## References

[B1] Abraldes J. G. Albillos A. Banares R. Turnes J. Gonzalez R. Garcia-Pagan J. C. (2009). Simvastatin lowers portal pressure in patients with cirrhosis and portal hypertension: a randomized controlled trial. *Gastroenterology* 136 1651–1658. 10.1053/j.gastro.2009.01.043 19208350

[B2] Abramowicz M. Zuccotti G. Pflomm J. M. (2017). Sofosbuvir/velpatasvir (epclusa) for hepatitis c. *JAMA* 317 639–640. 10.1001/jama.2016.12279 28196252

[B3] Akcora B. O. Storm G. Bansal R. (2018). Inhibition of canonical Wnt signaling pathway by beta-catenin/CBP inhibitor ICG-001 ameliorates liver fibrosis in vivo through suppression of stromal CXCL 12. *Biochim. Biophys. Acta Mol. Basis Dis.* 1864 804–818. 10.1016/j.bbadis.2017.12.001 29217140

[B4] Ameer F. Scandiuzzi L. Hasnain S. Kalbacher H. Zaidi N. (2014). De novo lipogenesis in health and disease. *Metabolism* 63 895–902. 10.1016/j.metabol.2014.04.003 24814684

[B5] Anstee Q. M. Neuschwander-Tetri B. A. Wong V. W. Abdelmalek M. F. Younossi Z. M. Yuan J. (2020). Cenicriviroc for the treatment of liver fibrosis in adults with nonalcoholic steatohepatitis: aurora phase 3 study design. *Contemp. Clin. Trials.* 89:105922. 10.1016/j.cct.2019.105922 31881392

[B6] Aoyama T. Paik Y. H. Watanabe S. Laleu B. Gaggini F. Fioraso-Cartier L. (2012). Nicotinamide adenine dinucleotide phosphate oxidase in experimental liver fibrosis: GKT137831 as a novel potential therapeutic agent. *Hepatology* 56 2316–2327. 10.1002/hep.25938 22806357 PMC3493679

[B7] Armstrong M. J. Gaunt P. Aithal G. P. Barton D. Hull D. Parker R. (2016). Liraglutide safety and efficacy in patients with non-alcoholic steatohepatitis (LEAN): a multicenter, double-blind, randomized, placebo-controlled phase 2 study. *Lancet* 387 679–690. 10.1016/S0140-6736(15)00803-X 26608256

[B8] Asrani S. K. Devarbhavi H. Eaton J. Kamath P. S. (2019). Burden of liver diseases in the world. *J. Hepatol.* 70 151–171. 10.1016/j.jhep.2018.09.014 30266282

[B9] Aydin M. M. Akcali K. C. (2018). Liver fibrosis. *Turk. J. Gastroenterol.* 29 14–21. 10.5152/tjg.2018.17330 29391303 PMC6322608

[B10] Barreyro F. J. Holod S. Finocchietto P. V. Camino A. M. Aquino J. B. Avagnina A. (2015). The pan-caspase inhibitor emricasan (IDN-6556) decreases liver injury and fibrosis in a murine model of non-alcoholic steatohepatitis. *Liver Int.* 35 953–966. 10.1111/liv.12570 24750664

[B11] Bataller R. Gao B. (2013). Dissecting the role of CB1 receptors on chronic liver diseases. *Gut* 62 957–958. 10.1136/gutjnl-2012-303664 23197412 PMC3779466

[B12] Benhamou Y. Bochet M. Di Martino V. Charlotte F. Azria F. Coutellier A. (1999). Liver fibrosis progression in human immunodeficiency virus and hepatitis C virus coinfected patients. *Multivirc. Group Hepatol.* 30 1054–1058. 10.1002/hep.510300409 10498659

[B13] Bernuth S. Yagmur E. Schuppan D. Sprinzl M. F. Zimmermann A. Schad A. (2016). Early changes in dynamic biomarkers of liver fibrosis in hepatitis C virus-infected patients treated with sofosbuvir. *Dig. Liver Dis.* 48 291–297. 10.1016/j.dld.2015.09.015 26514736

[B14] Berres M. L. Koenen R. R. Rueland A. Zaldivar M. M. Heinrichs D. Sahin H. (2010). Antagonism of the chemokine Ccl5 ameliorates experimental liver fibrosis in mice. *J. Clin. Invest.* 120 4129–4140. 10.1172/JCI41732 20978355 PMC2964968

[B15] Borkham-Kamphorst E. Weiskirchen R. (2016). The PDGF system and its antagonists in liver fibrosis. *Cytokine Growth Factor Rev.* 28 53–61. 10.1016/j.cytogfr.2015.10.002 26547628

[B16] Boyd A. Lasnier E. Molina J. M. Lascoux-Combe C. Bonnard P. Miailhes P. (2010). Liver fibrosis changes in HIV-HBV-coinfected patients: clinical, biochemical and histological effect of long-term tenofovir disoproxil fumarate use. *Antivir. Ther.* 15 963–974. 10.3851/IMP1649 21041911

[B17] Bruck R. Genina O. Aeed H. Alexiev R. Nagler A. Avni Y. (2001). Halofuginone to prevent and treat thioacetamide-induced liver fibrosis in rats. *Hepatology* 33 379–386. 10.1053/jhep.2001.21408 11172339

[B18] Buchman T. G. (2005). RNAi. *Crit. Care Med.* 33 S441–S443. 10.1097/01.ccm.0000191263.35901.5c 16340416

[B19] Campana L. Iredale J. P. (2017). Regression of liver fibrosis. *Semin. Liver Dis.* 37 1–10. 10.1055/s-0036-1597816 28201843

[B20] Chalasani N. Abdelmalek M. F. Garcia-Tsao G. Vuppalanchi R. Alkhouri N. Rinella M. (2020). Effects of belapectin, an inhibitor of galectin-3, in patients with nonalcoholic steatohepatitis with cirrhosis and portal hypertension. *Gastroenterology* 158 1334–1345. 10.1053/j.gastro.2019.11.296 31812510

[B21] Chen A. Zheng S. (2008). Curcumin inhibits connective tissue growth factor gene expression in activated hepatic stellate cells in vitro by blocking NF-kappaB and ERK signaling. *Br. J. Pharmacol.* 153 557–567. 10.1038/sj.bjp.0707542 17965732 PMC2241795

[B22] Chen Q. Chen L. Wu X. Zhang F. Jin H. Lu C. (2016). Dihydroartemisinin prevents liver fibrosis in bile duct ligated rats by inducing hepatic stellate cell apoptosis through modulating the PI3K/AKT pathway. *IUBMB Life* 68 220–231. 10.1002/iub.1478 26865509

[B23] Chen S. W. Wu B. Y. Xu S. P. Fan K. X. Yan L. Gong Y. (2012). Suppression of CB1 cannabinoid receptor by lentivirus mediated small interfering RNA ameliorates hepatic fibrosis in rats. *PLoS One* 7:e50850. 10.1371/journal.pone.0050850 23251393 PMC3520929

[B24] Cheng K. Yang N. Mahato R. I. (2009). TGF-beta1 gene silencing for treating liver fibrosis. *Mol. Pharm.* 6 772–779. 10.1021/mp9000469 19388665 PMC2743970

[B25] Cheng Q. Li C. Yang C. F. Zhong Y. J. Wu D. Shi L. (2019). Methyl ferulic acid attenuates liver fibrosis and hepatic stellate cell activation through the TGF-beta1/smad and NOX4/ROS pathways. *Chem. Biol. Interact.* 299 131–139. 10.1016/j.cbi.2018.12.006 30543783

[B26] Chhimwal J. Sharma S. Kulurkar P. Patial V. (2020). Crocin attenuates CCl4-induced liver fibrosis via PPAR-gamma mediated modulation of inflammation and fibrogenesis in rats. *Hum. Exp. Toxicol.* 39 1639–1649. 10.1177/0960327120937048 32633567

[B27] Cicha I. Zitzmann R. Goppelt-Struebe M. (2014). Dual inhibition of Src family kinases and aurora kinases by su6656 modulates CTGF (connective tissue growth factor) expression in an ERK-dependent manner. *Int. J. Biochem. Cell Biol*. 46 39–48. 10.1016/j.biocel.2013.11.014 24275091

[B28] Colmenero J. Bataller R. Sancho-Bru P. Dominguez M. Moreno M. Forns X. (2009). Effects of losartan on hepatic expression of nonphagocytic NADPH oxidase and fibrogenic genes in patients with chronic hepatitis C. *Am. J. Physiol. Gastrointest. Liver Physiol.* 297 G726–G734. 10.1152/ajpgi.00162.2009 19628656 PMC2763804

[B29] de Alwis N. M. Day C. P. (2008). Non-alcoholic fatty liver disease: the mist gradually clears. *J. Hepatol.* 48 S104–S112. 10.1016/j.jhep.2008.01.009 18304679

[B30] De Minicis S. Brenner D. A. (2007). NOX in liver fibrosis. *Arch. Biochem. Biophys.* 462 266–272. 10.1016/j.abb.2007.04.016 17531188 PMC2727549

[B31] Derynck R. Zhang Y. E. (2003). Smad-dependent and smad-independent pathways in TGF-beta family signaling. *Nature* 425 577–584. 10.1038/nature02006 14534577

[B32] Du G. Wang J. Zhang T. Ding Q. Jia X. Zhao X. (2020). Targeting Src family kinase member Fyn by saracatinib attenuated liver fibrosis in vitro and in vivo. *Cell Death Dis.* 11:118. 10.1038/s41419-020-2229-2 32051399 PMC7016006

[B33] Fan Y. Y. Ding W. Zhang C. Fu L. Xu D. X. Chen X. (2019). Obeticholic acid prevents carbon tetrachloride-induced liver fibrosis through interaction between farnesoid X receptor and smad3. *Int. Immunopharmacol.* 77:105911. 10.1016/j.intimp.2019.105911 31671330

[B34] Ferrell J. M. Pathak P. Boehme S. Gilliland T. Chiang J. (2019). Deficiency of both farnesoid X receptor and Takeda G protein-coupled receptor 5 exacerbated liver fibrosis in mice. *Hepatology* 70 955–970. 10.1002/hep.30513 30664797 PMC6642864

[B35] Flores-Contreras L. Sandoval-Rodriguez A. S. Mena-Enriquez M. G. Lucano-Landeros S. Arellano-Olivera I. Alvarez-Alvarez A. (2014). Treatment with pirfenidone for two years decreases fibrosis, cytokine levels and enhances CB2 gene expression in patients with chronic hepatitis C. *BMC Gastroenterol.* 14:131. 10.1186/1471-230X-14-131 25064094 PMC4236537

[B36] Frenette C. T. Morelli G. Shiffman M. L. Frederick R. T. Rubin R. A. Fallon M. B. (2019). Emricasan improves liver function in patients with cirrhosis and high model for end-stage liver disease scores compared with placebo. *Clin. Gastroenterol. Hepatol.* 17 774–783. 10.1016/j.cgh.2018.06.012 29913280

[B37] Friedman S. Sanyal A. Goodman Z. Lefebvre E. Gottwald M. Fischer L. (2016). Efficacy and safety study of cenicriviroc for the treatment of non-alcoholic steatohepatitis in adult subjects with liver fibrosis: centaur phase 2b study design. *Contemp. Clin. Trials.* 47 356–365. 10.1016/j.cct.2016.02.012 26944023

[B38] Friedman S. L. Ratziu V. Harrison S. A. Abdelmalek M. F. Aithal G. P. Caballeria J. (2018). A randomized, placebo-controlled trial of cenicriviroc for treatment of nonalcoholic steatohepatitis with fibrosis. *Hepatology* 67 1754–1767. 10.1002/hep.29477 28833331 PMC5947654

[B39] Gao Y. S. Qian M. Y. Wei Q. Q. Duan X. B. Wang S. L. Hu H. Y. (2020). WZ66, a novel acetyl-CoA carboxylase inhibitor, alleviates nonalcoholic steatohepatitis (NASH) in mice. *Acta Pharmacol. Sin.* 41 336–347. 10.1038/s41401-019-0310-0 31645659 PMC7468331

[B40] Garcia L. Hernandez I. Sandoval A. Salazar A. Garcia J. Vera J. (2002). Pirfenidone effectively reverses experimental liver fibrosis. *J. Hepatol.* 37 797–805. 10.1016/s0168-8278(02)00272-6 12445421

[B41] Ge W. S. Wang Y. J. Wu J. X. Fan J. G. Chen Y. W. Zhu L. (2014). Beta-catenin is overexpressed in hepatic fibrosis and blockage of Wnt/beta-catenin signaling inhibits hepatic stellate cell activation. *Mol. Med. Rep.* 9 2145–2151. 10.3892/mmr.2014.2099 24691643 PMC4055486

[B42] Giannone F. A. Baldassarre M. Domenicali M. Zaccherini G. Trevisani F. Bernardi M. (2012). Reversal of liver fibrosis by the antagonism of endocannabinoid CB1 receptor in a rat model of CCl(4)-induced advanced cirrhosis. *Lab. Invest.* 92 384–395. 10.1038/labinvest.2011.191 22184091

[B43] Goto T. Itoh M. Suganami T. Kanai S. Shirakawa I. Sakai T. (2018). Obeticholic acid protects against hepatocyte death and liver fibrosis in a murine model of nonalcoholic steatohepatitis. *Sci. Rep.* 8:8157. 10.1038/s41598-018-26383-8 29802399 PMC5970222

[B44] Gracia-Sancho J. Manicardi N. Ortega-Ribera M. Maeso-Diaz R. Guixe-Muntet S. Fernandez-Iglesias A. (2019). Emricasan ameliorates portal hypertension and liver fibrosis in cirrhotic rats through a hepatocyte-mediated paracrine mechanism. *Hepatol. Commun.* 3 987–1000. 10.1002/hep4.1360 31304452 PMC6601324

[B45] Gudowska M. Gruszewska E. Cylwik B. Panasiuk A. Rogalska M. Flisiak R. (2015). Galectin-3 concentration in liver diseases. *Ann. Clin. Lab. Sci.* 45 669–673. 26663797

[B46] Harrison S. A. Bashir M. R. Guy C. D. Zhou R. Moylan C. A. Frias J. P. (2019). Resmetirom (MGL-3196) for the treatment of non-alcoholic steatohepatitis: a multicenter, randomized, double-blind, placebo-controlled, phase 2 trial. *Lancet* 394 2012–2024. 10.1016/S0140-6736(19)32517-6 31727409

[B47] Harrison S. A. Goodman Z. Jabbar A. Vemulapalli R. Younes Z. H. Freilich B. (2020a). A randomized, placebo-controlled trial of emricasan in patients with NASH and F1-F3 fibrosis. *J. Hepatol.* 72 816–827. 10.1016/j.jhep.2019.11.024 31887369

[B48] Harrison S. A. Wong V. W. Okanoue T. Bzowej N. Vuppalanchi R. Younes Z. (2020c). Selonsertib for patients with bridging fibrosis or compensated cirrhosis due to NASH: results from randomized phase III stellar trials. *J. Hepatol.* 73 26–39. 10.1016/j.jhep.2020.02.027 32147362

[B49] Harrison S. A. Rossi S. J. Paredes A. H. Trotter J. F. Bashir M. R. Guy C. D. (2020b). NGM282 improves liver fibrosis and histology in 12 weeks in patients with nonalcoholic steatohepatitis. *Hepatology* 71 1198–1212. 10.1002/hep.30590 30805949 PMC7187438

[B50] Harrison S. A. Marri S. R. Chalasani N. Kohli R. Aronstein W. Thompson G. A. (2016). Randomised clinical study: GR-MD-02, a galectin-3 inhibitor, vs. Placebo in patients having non-alcoholic steatohepatitis with advanced fibrosis. *Aliment. Pharmacol. Ther.* 44 1183–1198. 10.1111/apt.13816 27778367

[B51] Harrison S. A. Rinella M. E. Abdelmalek M. F. Trotter J. F. Paredes A. H. Arnold H. L. (2018). NGM282 for treatment of non-alcoholic steatohepatitis: a multicenter, randomized, double-blind, placebo-controlled, phase 2 trial. *Lancet* 391 1174–1185. 10.1016/S0140-6736(18)30474-4 29519502

[B52] Iredale J. P. Thompson A. Henderson N. C. (2013). Extracellular matrix degradation in liver fibrosis: biochemistry and regulation. *Biochim. Biophys. Acta* 1832 876–883. 10.1016/j.bbadis.2012.11.002 23149387

[B53] Iwasaki T. Yoneda M. Inamori M. Shirakawa J. Higurashi T. Maeda S. (2011). Sitagliptin as a novel treatment agent for non-alcoholic fatty liver disease patients with type 2 diabetes mellitus. *Hepatogastroenterology* 58 2103–2105. 10.5754/hge11263 22024083

[B54] Jang Y. O. Kim S. H. Cho M. Y. Kim K. S. Park K. S. Cha S. K. (2018). Synergistic effects of simvastatin and bone marrow-derived mesenchymal stem cells on hepatic fibrosis. *Biochem. Biophys. Res. Commun.* 497 264–271. 10.1016/j.bbrc.2018.02.067 29428718

[B55] Jia J. B. Liu Y. Chen W. H. Liu M. Lu L. G. (2007). [Effects of pioglitazone on the morphology and the expression of connective tissue growth factor of transforming growth factor beta-induced rat hepatic stellate cells in vitro]. *Zhonghua Gan Zang Bing Za Zhi* 15 192–195. 17407709

[B56] Jiang J. X. Chen X. Serizawa N. Szyndralewiez C. Page P. Schroder K. (2012). Liver fibrosis and hepatocyte apoptosis are attenuated by GKT137831, a novel NOX4/NOX1 inhibitor in vivo. *Free Radic. Biol. Med.* 53 289–296. 10.1016/j.freeradbiomed.2012.05.007 22618020 PMC3392471

[B57] Jimenez C. C. Sehgal A. Popov Y. Kim Y. O. Zevallos V. Sahin U. (2015). Specific hepatic delivery of procollagen alpha1(I) small interfering RNA in lipid-like nanoparticles resolves liver fibrosis. *Hepatology* 62 1285–1297. 10.1002/hep.27936 26096209 PMC4589454

[B58] Kavita U. Miller W. Ji Q. C. Pillutla R. C. (2019). A fit-for-purpose method for the detection of human antibodies to surface-exposed components of BMS-986263, a lipid nanoparticle-based drug product containing a siRNA drug substance. *AAPS J.* 21:92. 10.1208/s12248-019-0360-8 31332587

[B59] Kimura K. Ikoma A. Shibakawa M. Shimoda S. Harada K. Saio M. (2017). Safety, tolerability, and preliminary efficacy of the anti-fibrotic small molecule PRI-724, a CBP/beta-catenin inhibitor, in patients with hepatitis C virus-related cirrhosis: a single-center, open-label, dose escalation phase 1 trial. *E Bio Med.* 23 79–87. 10.1016/j.ebiom.2017.08.016 28844410 PMC5605374

[B60] Kodai S. Takemura S. Kubo S. Azuma H. Minamiyama Y. (2015). Therapeutic administration of an ingredient of aged-garlic extracts, s-allyl cysteine resolves liver fibrosis established by carbon tetrachloride in rats. *J. Clin. Biochem. Nutr.* 56 179–185. 10.3164/jcbn.14-108 26060347 PMC4454081

[B61] Kovalenko E. Tacke F. Gressner O. A. Zimmermann H. W. Lahme B. Janetzko A. (2009). Validation of connective tissue growth factor (CTGF/CCN2) and its gene polymorphisms as noninvasive biomarkers for the assessment of liver fibrosis. *J. Viral Hepat.* 16 612–620. 10.1111/j.1365-2893.2009.01110.x 19243500

[B62] Lefebvre E. Moyle G. Reshef R. Richman L. P. Thompson M. Hong F. (2016). Antifibrotic effects of the dual CCR2/CCR5 antagonist cenicriviroc in animal models of liver and kidney fibrosis. *PLoS One* 11:e158156. 10.1371/journal.pone.0158156 27347680 PMC4922569

[B63] Lei Y. Wang Q. L. Shen L. Tao Y. Y. Liu C. H. (2019). MicroRNA-101 suppresses liver fibrosis by downregulating PI3K/AKT/mTOR signaling pathway. *Clin. Res. Hepatol. Gastroenterol.* 43 575–584. 10.1016/j.clinre.2019.02.003 30857885

[B64] Li G. M. Shi Y. Li D. G. Xie Q. Guo Q. Jin Y. X. (2004). [Effect of small interfering RNA targeting connective tissue growth factor on the synthesis and secretion of extracellular matrix in hepatic stellate cells]. *Zhonghua Gan Zang Bing Za Zhi* 12 526–529. 15387902

[B65] Li L. Wang B. E. (2007). [Kupffer cells and liver fibrosis]. *Zhonghua Gan Zang Bing Za Zhi* 15 559–560. 17669258

[B66] Li M. O. Wan Y. Y. Sanjabi S. Robertson A. K. Flavell R. A. (2006). Transforming growth factor-beta regulation of immune responses. *Annu. Rev. Immunol.* 24 99–146. 10.1146/annurev.immunol.24.021605.090737 16551245

[B67] Li X. Wu X. Q. Xu T. Li X. F. Yang Y. Li W. X. (2016). Role of histone deacetylases (HDACs) in progression and reversal of liver fibrosis. *Toxicol. Appl. Pharmacol.* 306 58–68. 10.1016/j.taap.2016.07.003 27396813

[B68] Liang J. Zhang B. Shen R. W. Liu J. B. Gao M. H. Li Y. (2013). Preventive effect of halofuginone on concanavalin a-induced liver fibrosis. *PLoS One* 8:e82232. 10.1371/journal.pone.0082232 24358159 PMC3864948

[B69] Lim B. J. Lee W. Lee H. W. Lee K. S. Kim J. K. Chang H. Y. (2018). Selective deletion of hepatocyte platelet-derived growth factor receptor α and development of liver fibrosis in mice. *Cell Commun. Signal.* 16:93. 10.1186/s12964-018-0306-2 30509307 PMC6276164

[B70] Link J. O. Taylor J. G. Trejo-Martin A. Kato D. Katana A. A. Krygowski E. S. (2019). Discovery of velpatasvir (GS-5816): a potent pan-genotypic HCV NS5A inhibitor in the single-tablet regimens vosevi and epclusa. *Bioorg. Med. Chem. Lett.* 29 2415–2427. 10.1016/j.bmcl.2019.04.027 31230974

[B71] Liu J. Kong D. Qiu J. Xie Y. Lu Z. Zhou C. (2019). Praziquantel ameliorates CCl4-induced liver fibrosis in mice by inhibiting TGF-beta/smad signaling via up-regulating smad7 in hepatic stellate cells. *Br. J. Pharmacol.* 176 4666–4680. 10.1111/bph.14831 31412137 PMC6965681

[B72] Liu J. Zhang Z. Gao J. Xie J. Yang L. Hu S. (2011). Downregulation effects of beta-elemene on the levels of plasma endotoxin, serum TNF-alpha, and hepatic CD14 expression in rats with liver fibrosis. *Front. Med.* 5:101–105. 10.1007/s11684-011-0111-4 21681682

[B73] Liu Y. Nong L. Jia Y. Tan A. Duan L. Lu Y. (2020). Aspirin alleviates hepatic fibrosis by suppressing hepatic stellate cells activation via the TLR4/NF-kappaB pathway. *Aging (Albany N. Y.)* 12 6058–6066. 10.18632/aging.103002 32283542 PMC7185140

[B74] Liu Y. Wu J. Li Z. Luo Y. Zeng F. Shi S. (2017). Tolerability and pharmacokinetics of hydronidone, an antifibrotic agent for hepatic fibrosis, after single and multiple doses in healthy subjects: an open-label, randomized, dose-escalating, first-in-human study. *Eur. J. Drug Metab. Pharmacokinet.* 42 37–48. 10.1007/s13318-015-0316-z 26797810

[B75] Loomba R. Kayali Z. Noureddin M. Ruane P. Lawitz E. J. Bennett M. (2018a). GS-0976 reduces hepatic steatosis and fibrosis markers in patients with nonalcoholic fatty liver disease. *Gastroenterology* 155 1463–1473. 10.1053/j.gastro.2018.07.027 30059671 PMC6318218

[B76] Loomba R. Lawitz E. Mantry P. S. Jayakumar S. Caldwell S. H. Arnold H. (2018b). The ASK1 inhibitor selonsertib in patients with nonalcoholic steatohepatitis: a randomized, phase 2 trial. *Hepatology* 67 549–559. 10.1002/hep.29514 28892558 PMC5814892

[B77] Luo W. Xu Q. Wang Q. Wu H. Hua J. (2017). Effect of modulation of PPAR-gamma activity on kupffer cells M1/M2 polarization in the development of non-alcoholic fatty liver disease. *Sci. Rep.* 7:44612. 10.1038/srep44612 28300213 PMC5353732

[B78] Marti-Rodrigo A. Alegre F. Moragrega A. B. Garcia-Garcia F. Marti-Rodrigo P. Fernandez-Iglesias A. (2020). Rilpivirine attenuates liver fibrosis through selective stat1-mediated apoptosis in hepatic stellate cells. *Gut* 69 920–932. 10.1136/gutjnl-2019-318372 31530714

[B79] Meier R. Meyer J. Montanari E. Lacotte S. Balaphas A. Muller Y. (2019). Interleukin-1 receptor antagonist modulates liver inflammation and fibrosis in mice in a model-dependent manner. *Int. J. Mol. Sci.* 20:1295. 10.3390/ijms20061295 30875826 PMC6471711

[B80] Meissner E. G. McLaughlin M. Matthews L. Gharib A. M. Wood B. J. Levy E. (2016). Simtuzumab treatment of advanced liver fibrosis in HIV and HCV-infected adults: results of a 6-month open-label safety trial. *Liver Int.* 36 1783–1792. 10.1111/liv.13177 27232579 PMC5116256

[B81] Mihm S. (2018). Danger-associated molecular patterns (DAMPs): molecular triggers for sterile inflammation in the liver. *Int. J. Mol. Sci.* 19:3104. 10.3390/ijms19103104 30309020 PMC6213769

[B82] Moon H. W. Park M. Hur M. Kim H. Choe W. H. Yun Y. M. (2018). Usefulness of enhanced liver fibrosis, glycosylation isomer of MAC-2 binding protein, galectin-3, and soluble suppression of tumorigenicity 2 for assessing liver fibrosis in chronic liver diseases. *Ann. Lab. Med.* 38 331–337. 10.3343/alm.2018.38.4.331 29611383 PMC5895862

[B83] Mortezaee K. (2018). Nicotinamide adenine dinucleotide phosphate (NADPH) oxidase (NOX) and liver fibrosis: a review. *Cell Biochem. Funct.* 36 292–302. 10.1002/cbf.3351 30028028

[B84] Mu M. Zuo S. Wu R. M. Deng K. S. Lu S. Zhu J. J. (2018). Ferulic acid attenuates liver fibrosis and hepatic stellate cell activation via inhibition of TGF-beta/smad signaling pathway. *Drug Des. Devel. Ther.* 12 4107–4115. 10.2147/DDDT.S186726 30584275 PMC6284527

[B85] Mueller S. Millonig G. Sarovska L. Friedrich S. Reimann F. M. Pritsch M. (2010). Increased liver stiffness in alcoholic liver disease: differentiating fibrosis from steatohepatitis. *World J. Gastroenterol.* 16 966–972. 10.3748/wjg.v16.i8.966 20180235 PMC2828601

[B86] Neuschwander-Tetri B. A. Loomba R. Sanyal A. J. Lavine J. E. Van Natta M. L. Abdelmalek M. F. (2015). Farnesoid X nuclear receptor ligand obeticholic acid for non-cirrhotic, non-alcoholic steatohepatitis (FLINT): a multicenter, randomized, placebo-controlled trial. *Lancet* 385 956–965. 10.1016/S0140-6736(14)61933-4 25468160 PMC4447192

[B87] Nishikawa K. Osawa Y. Kimura K. (2018). Wnt/beta-catenin signaling as a potential target for the treatment of liver cirrhosis using antifibrotic drugs. *Int. J. Mol. Sci.* 19:3103. 10.3390/ijms19103103 30308992 PMC6213128

[B88] Oberti F. Pilette C. Rifflet H. Maiga M. Y. Moreau A. Gallois Y. (1997). Effects of simvastatin, pentoxifylline and spironolactone on hepatic fibrosis and portal hypertension in rats with bile duct ligation. *J. Hepatol.* 26 1363–1371. 10.1016/s0168-8278(97)80473-4 9210625

[B89] Patel K. Harrison S. A. Elkhashab M. Trotter J. F. Herring R. Rojter S. E. (2020). Cilofexor, a nonsteroidal FXR agonist, in patients with noncirrhotic NASH: a phase 2 randomized controlled trial. *Hepatology* 72 58–71. 10.1002/hep.31205 32115759

[B90] Pedrosa M. Seyedkazemi S. Francque S. Sanyal A. Rinella M. Charlton M. (2020). A randomized, double-blind, multicenter, phase 2b study to evaluate the safety and efficacy of a combination of tropifexor and cenicriviroc in patients with nonalcoholic steatohepatitis and liver fibrosis: study design of the tandem trial. *Contemp. Clin. Trials* 88:105889. 10.1016/j.cct.2019.105889 31731005

[B91] Peng W. H. Tien Y. C. Huang C. Y. Huang T. H. Liao J. C. Kuo C. L. (2010). Fraxinus rhynchophylla ethanol extract attenuates carbon tetrachloride-induced liver fibrosis in rats via down-regulating the expressions of uPA, MMP-2, MMP-9 and TIMP-1. *J. Ethnopharmacol.* 127 606–613. 10.1016/j.jep.2009.12.016 20035854

[B92] Peng Y. Li L. Zhang X. Xie M. Yang C. Tu S. (2019). Fluorofenidone affects hepatic stellate cell activation in hepatic fibrosis by targeting the TGF-beta1/smad and MAPK signaling pathways. *Exp. Ther. Med.* 18 41–48. 10.3892/etm.2019.7548 31258636 PMC6566051

[B93] Perepelyuk M. Terajima M. Wang A. Y. Georges P. C. Janmey P. A. Yamauchi M. (2013). Hepatic stellate cells and portal fibroblasts are the major cellular sources of collagens and lysyl oxidases in normal liver and early after injury. *Am. J. Physiol. Gastrointest. Liver Physiol.* 304 G605–G614. 10.1152/ajpgi.00222.2012 23328207 PMC3602686

[B94] Pinzani M. Macias-Barragan J. (2010). Update on the pathophysiology of liver fibrosis. *Expert. Rev. Gastroenterol. Hepatol.* 4 459–472. 10.1586/egh.10.47 20678019

[B95] Poo J. L. Torre A. Aguilar-Ramirez J. R. Cruz M. Mejia-Cuan L. Cerda E. (2020). Benefits of prolonged-release pirfenidone plus standard of care treatment in patients with advanced liver fibrosis: PROMETEO study. *Hepatol. Int.* 14 817–827. 10.1007/s12072-020-10069-3 32813194 PMC7561536

[B96] Povero D. Busletta C. Novo E. di Bonzo L. V. Cannito S. Paternostro C. (2010). Liver fibrosis: a dynamic and potentially reversible process. *Histol. Histopathol.* 25 1075–1091. 10.14670/HH-25.1075 20552556

[B97] Poynard T. Bedossa P. Opolon P. (1997). Natural history of liver fibrosis progression in patients with chronic hepatitis C. The obsvirc, metavir, clinivir, and dosvirc groups. *Lancet* 349 825–832. 10.1016/s0140-6736(96)07642-8 9121257

[B98] Premkumar M. Dhiman R. K. (2018). Direct-acting antiviral agents for HCV infection. *J. Clin. Exp. Hepatol.* 8 1–2. 10.1016/j.jceh.2018.01.002 29743789 PMC5938327

[B99] Rallón N. I. Barreiro P. Soriano V. García-Samaniego J. López M. Benito J. M. (2011). Elevated TGF-β1 levels might protect HCV/HIV-coinfected patients from liver fibrosis. *Eur. J. Clin. Invest.* 41 70–76. 10.1111/j.1365-2362.2010.02381.x 20868448

[B100] Ratziu V. Harrison S. A. Francque S. Bedossa P. Lehert P. Serfaty L. (2016). Elafibranor, an agonist of the peroxisome proliferator-activated receptor-alpha and -delta, induces resolution of nonalcoholic steatohepatitis without fibrosis worsening. *Gastroenterology* 150 1147–1159. 10.1053/j.gastro.2016.01.038 26874076

[B101] Ratziu V. Sanyal A. J. Loomba R. Rinella M. Harrison S. Anstee Q. M. (2019). Regenerate: design of a pivotal, randomized, phase 3 study evaluating the safety and efficacy of obeticholic acid in patients with fibrosis due to nonalcoholic steatohepatitis. *Contemp. Clin. Trials* 84:105803. 10.1016/j.cct.2019.06.017 31260793

[B102] Roehlen N. Crouchet E. Baumert T. F. (2020). Liver fibrosis: mechanistic concepts and therapeutic perspectives. *Cells* 9:875. 10.3390/cells9040875 32260126 PMC7226751

[B103] Ross T. T. Crowley C. Kelly K. L. Rinaldi A. Beebe D. A. Lech M. P. (2020). Acetyl-CoA carboxylase inhibition improves multiple dimensions of NASH pathogenesis in model systems. *Cell Mol. Gastroenterol. Hepatol.* 10 829–851. 10.1016/j.jcmgh.2020.06.001 32526482 PMC7509217

[B104] Salah M. M. Ashour A. A. Abdelghany T. M. Abdel-Aziz A. H. Salama S. A. (2019). Pirfenidone alleviates concanavalin a-induced liver fibrosis in mice. *Life Sci.* 239:116982. 10.1016/j.lfs.2019.116982 31639402

[B105] Sanyal A. J. Charles E. D. Neuschwander-Tetri B. A. Loomba R. Harrison S. A. Abdelmalek M. F. (2019a). Pegbelfermin (BMS-986036), a pegylated fibroblast growth factor 21 analogue, in patients with non-alcoholic steatohepatitis: a randomized, double-blind, placebo-controlled, phase 2a trial. *Lancet* 392 2705–2717. 10.1016/S0140-6736(18)31785-9 30554783

[B106] Sanyal A. J. Harrison S. A. Ratziu V. Abdelmalek M. F. Diehl A. M. Caldwell S. (2019b). The natural history of advanced fibrosis due to nonalcoholic steatohepatitis: data from the simtuzumab trials. *Hepatology* 70 1913–1927. 10.1002/hep.30664 30993748

[B107] Sato Y. Murase K. Kato J. Kobune M. Sato T. Kawano Y. (2008). Resolution of liver cirrhosis using vitamin a-coupled liposomes to deliver siRNA against a collagen-specific chaperone. *Nat. Biotechnol.* 26 431–442. 10.1038/nbt1396 18376398

[B108] Schwabe R. F. Brenner D. A. (2006). Mechanisms of liver injury. I. TNF-alpha-induced liver injury: role of IKK, JNK, and ROS pathways. *Am. J. Physiol. Gastrointest. Liver Physiol.* 290 G583–G589. 10.1152/ajpgi.00422.2005 16537970

[B109] Schwabl P. Hambruch E. Seeland B. A. Hayden H. Wagner M. Garnys L. (2017). The FXR agonist PX20606 ameliorates portal hypertension by targeting vascular remodeling and sinusoidal dysfunction. *J. Hepatol.* 66 724–733. 10.1016/j.jhep.2016.12.005 27993716

[B110] Seniutkin O. Furuya S. Luo Y. S. Cichocki J. A. Fukushima H. Kato Y. (2018). Effects of pirfenidone in acute and sub-chronic liver fibrosis, and an initiation-promotion cancer model in the mouse. *Toxicol. Appl. Pharmacol.* 339 1–9. 10.1016/j.taap.2017.11.024 29197520

[B111] Seo H. Y. Lee S. H. Lee J. H. Kang Y. N. Hwang J. S. Park K. G. (2020). Src inhibition attenuates liver fibrosis by preventing hepatic stellate cell activation and decreasing connetive tissue growth factor. *Cells* 9:558. 10.3390/cells9030558 32120837 PMC7140470

[B112] Shen M. Guo M. Wang Z. Li Y. Kong D. Shao J. (2020). ROS-dependent inhibition of the PI3K/AKT/mTOR signaling is required for oroxylin a to exert anti-inflammatory activity in liver fibrosis. *Int. Immunopharmacol.* 85:106637. 10.1016/j.intimp.2020.106637 32512269

[B113] Shiha G. E. Abu-Elsaad N. M. Zalata K. R. Ibrahim T. M. (2014). Tracking anti-fibrotic pathways of nilotinib and imatinib in experimentally induced liver fibrosis: an insight. *Clin. Exp. Pharmacol. Physiol.* 41 788–797. 10.1111/1440-1681.12286 25115651

[B114] Sun K. Huang X. H. Wang Q. (2009). [Peroxisome proliferator-activated receptor gamma inhibits transforming growth factor beta1-induced connective tissue growth factor expression in rat hepatic stellate cells]. *Nan Fang Yi Ke Da Xue Xue Bao* 29 1354–1358. 19620052

[B115] Sung Y. C. Liu Y. C. Chao P. H. Chang C. C. Jin P. R. Lin T. T. (2018). Combined delivery of sorafenib and a MEK inhibitor using CXCR4-targeted nanoparticles reduces hepatic fibrosis and prevents tumor development. *Theranostics* 8 894–905. 10.7150/thno.21168 29463989 PMC5817100

[B116] Tacke F. (2018). Cenicriviroc for the treatment of non-alcoholic steatohepatitis and liver fibrosis. *Expert Opin. Inv. Drug* 27 301–311. 10.1080/13543784.2018.1442436 29448843

[B117] Tacke F. Weiskirchen R. (2012). Update on hepatic stellate cells: pathogenic role in liver fibrosis and novel isolation techniques. *Expert Rev. Gastroenterol. Hepatol.* 6 67–80. 10.1586/egh.11.92 22149583

[B118] Tacke F. Weiskirchen R. (2021). Non-alcoholic fatty liver disease (NAFLD)/non-alcoholic steatohepatitis (NASH)-related liver fibrosis: mechanisms, treatment and prevention. *Ann. Transl. Med.* 9:729. 10.21037/atm-20-4354 33987427 PMC8106094

[B119] Tamura S. Shimomura I. (2005). Contribution of adipose tissue and de novo lipogenesis to nonalcoholic fatty liver disease. *J. Clin. Invest.* 115 1139–1142. 10.1172/JCI24930 15864343 PMC1087181

[B120] Tan S. Liu H. Ke B. Jiang J. Wu B. (2020). The peripheral CB1 receptor antagonist JD5037 attenuates liver fibrosis via a CB1 receptor/beta-arrestin1/AKT pathway. *Br. J. Pharmacol.* 177 2830–2847. 10.1111/bph.15010 32017042 PMC7236068

[B121] Teixeira-Clerc F. Julien B. Grenard P. Tran V. N. J. Deveaux V. Li L. (2006). CB1 cannabinoid receptor antagonism: a new strategy for the treatment of liver fibrosis. *Nat. Med.* 12 671–676. 10.1038/nm1421 16715087

[B122] Toda K. Kumagai N. Kaneko F. Tsunematsu S. Tsuchimoto K. Saito H. (2009). Pentoxifylline prevents pig serum-induced rat liver fibrosis by inhibiting interleukin-6 production. *J. Gastroenterol. Hepatol.* 24 860–865. 10.1111/j.1440-1746.2008.05749.x 19220679

[B123] Tokunaga Y. Osawa Y. Ohtsuki T. Hayashi Y. Yamaji K. Yamane D. (2017). Selective inhibitor of Wnt/beta-catenin/CBP signaling ameliorates hepatitis C virus-induced liver fibrosis in mouse model. *Sci. Rep.* 7:325. 10.1038/s41598-017-00282-w 28336942 PMC5427997

[B124] Valera J. M. Smok G. Marquez S. Poniachik J. Brahm J. (2011). [Histological regression of liver fibrosis with immunosuppressive therapy in autoimmune hepatitis]. *Gastroenterol. Hepatol.* 34 10–15. 10.1016/j.gastrohep.2010.10.003 21194803

[B125] Verzijl C. Van De Peppel I. P. Struik D. Jonker J. W. (2020). Pegbelfermin (BMS-986036): an investigational pegylated fibroblast growth factor 21 analogue for the treatment of nonalcoholic steatohepatitis. *Expert Opin. Investig. Drugs* 29 125–133. 10.1080/13543784.2020.1708898 31899984

[B126] Wan M. Xu H. Li D. Wang L. Li X. (2021). Accuracy of gamma-glutamyl transpeptidase-to-platelet ratio (GPR), red cell distribution width (RDW), aspartate aminotransferase-to-platelet ratio index (APRI), and the fibrosis-4 index (FIB4) compared with liver biopsy in patients with drug-induced liver injury (DIDL). *Medicine (Baltimore)* 100:e24723. 10.1097/MD.0000000000024723 33578617 PMC10545076

[B127] Wang H. Ge C. Zhou J. Guo Y. Cui S. Huang N. (2018). Noncanonical farnesoid X receptor signaling inhibits apoptosis and impedes liver fibrosis. *E Bio Med.* 37 322–333. 10.1016/j.ebiom.2018.10.028 30337250 PMC6286639

[B128] Wang J. Chu E. S. Chen H. Y. Man K. Go M. Y. Huang X. R. (2015). Microrna-29b prevents liver fibrosis by attenuating hepatic stellate cell activation and inducing apoptosis through targeting PI3K/AKT pathway. *Oncotarget* 6 7325–7338. 10.18632/oncotarget.2621 25356754 PMC4466688

[B129] Wang Y. Gao J. Zhang D. Zhang J. Ma J. Jiang H. (2010). New insights into the antifibrotic effects of sorafenib on hepatic stellate cells and liver fibrosis. *J. Hepatol.* 53 132–144. 10.1016/j.jhep.2010.02.027 20447716

[B130] Wei L. Chen Q. Guo A. Fan J. Wang R. Zhang H. (2018). Asiatic acid attenuates ccl4-induced liver fibrosis in rats by regulating the PI3K/AKT/mTOR and Bcl-2/Bax signaling pathways. *Int. Immunopharmacol.* 60 1–8. 10.1016/j.intimp.2018.04.016 29702278

[B131] Wei Z. Zhao D. Zhang Y. Chen Y. Zhang S. Li Q. (2019). Rosiglitazone ameliorates bile duct ligation-induced liver fibrosis by down-regulating NF-kappaB-TNF-alpha signaling pathway in a PPARgamma-dependent manner. *Biochem. Biophys. Res. Commun.* 519 854–860. 10.1016/j.bbrc.2019.09.084 31561855

[B132] Witek R. P. Stone W. C. Karaca F. G. Syn W. K. Pereira T. A. Agboola K. M. (2009). Pan-caspase inhibitor VX-166 reduces fibrosis in an animal model of nonalcoholic steatohepatitis. *Hepatology* 50 1421–1430. 10.1002/hep.23167 19676126

[B133] Wu S. D. Liu L. L. Cheng J. L. Liu Y. Cheng L. S. Wang S. Q. (2018). Longitudinal monitoring of liver fibrosis status by transient elastography in chronic hepatitis b patients during long-term entecavir treatment. *Clin. Exp. Med.* 18 433–443. 10.1007/s10238-018-0501-x 29696462

[B134] Xu H. Hong S. Yan Z. Zhao Q. Shi Y. Song N. (2019). RAP-8 ameliorates liver fibrosis by modulating cell cycle and oxidative stress. *Life Sci.* 229 200–209. 10.1016/j.lfs.2019.04.037 31047894

[B135] Xu P. Zhang Y. Liu Y. Yuan Q. Song L. Liu M. (2016). Fibroblast growth factor 21 attenuates hepatic fibrogenesis through TGF-beta/smad2/3 and NF-kappaB signaling pathways. *Toxicol. Appl. Pharmacol.* 290 43–53. 10.1016/j.taap.2015.11.012 26592322

[B136] Yang K. L. Chang W. T. Hong M. Y. Hung K. C. Chuang C. C. (2017). Prevention of TGF-beta-induced early liver fibrosis by a maleic acid derivative anti-oxidant through suppression of ROS, inflammation and hepatic stellate cells activation. *PLoS One*. 12:e174008. 10.1371/journal.pone.0174008 28384213 PMC5383026

[B137] Yoon Y. C. Fang Z. Lee J. E. Park J. H. Ryu J. K. Jung K. H. (2020). Selonsertib inhibits liver fibrosis via downregulation of ASK1/MAPK pathway of hepatic stellate cells. *Biomol. Ther. (Seoul.)* 28 527–536. 10.4062/biomolther.2020.016 32451370 PMC7585640

[B138] Younossi Z. M. Ratziu V. Loomba R. Rinella M. Anstee Q. M. Goodman Z. (2019). Obeticholic acid for the treatment of non-alcoholic steatohepatitis: interim analysis from a multicenter, randomized, placebo-controlled phase 3 trial. *Lancet* 394 2184–2196. 10.1016/S0140-6736(19)33041-7 31813633

[B139] Younossi Z. M. Stepanova M. Nader F. Loomba R. Anstee Q. M. Ratziu V. (2021). Obeticholic acid impact on quality of life in patients with nonalcoholic steatohepatitis: regenerate 18-month interim analysis. *Clin. Gastroenterol. Hepatol.* 21 00751–00755. 10.1016/j.cgh.2021.07.020 34274514

[B140] Zeng C. Wang Y. L. Xie C. Sang Y. Li T. J. Zhang M. (2015). Identification of a novel TGF-beta-miR-122-fibronectin 1/serum response factor signaling cascade and its implication in hepatic fibrogenesis. *Oncotarget* 6 12224–12233. 10.18632/oncotarget.3652 25909171 PMC4494934

[B141] Zhang C. Liu X. Q. Sun H. N. Meng X. M. Bao Y. W. Zhang H. P. (2018). Octreotide attenuates hepatic fibrosis and hepatic stellate cells proliferation and activation by inhibiting Wnt/beta-catenin signaling pathway, c-Myc and cyclin D1. *Int. Immunopharmacol.* 63 183–190. 10.1016/j.intimp.2018.08.005 30098497

[B142] Zhang H. Sun D. Wang G. Cui S. Field R. A. Li J. (2019). Alogliptin alleviates liver fibrosis via suppression of activated hepatic stellate cell. *Biochem. Biophys. Res. Co* 511 387–393. 10.1016/j.bbrc.2019.02.065 30797555

[B143] Zhang X. Arnott J. A. Rehman S. Delong W. J. Sanjay A. Safadi F. F. (2010). Src is a major signaling component for CTGF induction by TGF-beta1 in osteoblasts. *J. Cell. Physiol.* 224 691–701. 10.1002/jcp.22173 20432467 PMC2897974

[B144] Zhu Y. K. Wang B. E. Shen F. J. Wang A. M. Jia J. D. Ma H. (2004). [Dynamic evolution of MMP-13, TIMP-1, type I and III collagen and their interaction in experimental liver fibrosis]. *Zhonghua Gan Zang Bing Za Zhi* 12 612–615. 15504294

[B145] Zoubek M. E. Trautwein C. Strnad P. (2017). Reversal of liver fibrosis: from fiction to reality. *Best Pract. Res. Clin. Gastroenterol.* 31 129–141. 10.1016/j.bpg.2017.04.005 28624101

